# Differential regulation of cysteine oxidative post-translational modifications in high and low aerobic capacity

**DOI:** 10.1038/s41598-018-35728-2

**Published:** 2018-12-11

**Authors:** Rodrigo W. A. Souza, Christiano R. R. Alves, Alessandra Medeiros, Natale Rolim, Gustavo J. J. Silva, José B. N. Moreira, Marcia N. Alves, Martin Wohlwend, Mohammed Gebriel, Lars Hagen, Animesh Sharma, Lauren G. Koch, Steven L. Britton, Geir Slupphaug, Ulrik Wisløff, Patricia C. Brum

**Affiliations:** 1 School of Physical Education and Sport, University of São Paulo, São Paulo, Brazil; 2 Section on Integrative Physiology and Metabolism, Joslin Diabetes Center, Harvard Medical School, Boston, Massachusetts USA; 3 Biosciences Department, Federal University of São Paulo, Santos, Brazil; 4 K.G. Jebsen Center of Exercise in Medicine, Department of Circulation and Medical Imaging, Norwegian University of Science and Technology (NTNU), Trondheim, Norway; 5 Department of Cancer Research and Molecular Medicine and PROMEC Core Facility for Proteomics and Modomics, Norwegian University of Science and Technology (NTNU), and Central Norway Regional Health Authority, Trondheim, Norway; 6 Department of Physiology & Pharmacology, The University of Toledo, Toledo, Ohio USA; 7 Department of Anesthesiology, University of Michigan - Medical School, Ann Arbor, Michigan USA; 8 Department of Molecular and Integrative Physiology, University of Michigan, Ann Arbor, Michigan USA; 9 School of Human Movement & Nutrition Sciences, University of Queensland, Brisbane, Australia

## Abstract

Given the association between high aerobic capacity and the prevention of metabolic diseases, elucidating the mechanisms by which high aerobic capacity regulates whole-body metabolic homeostasis is a major research challenge. Oxidative post-translational modifications (Ox-PTMs) of proteins can regulate cellular homeostasis in skeletal and cardiac muscles, but the relationship between Ox-PTMs and intrinsic components of oxidative energy metabolism is still unclear. Here, we evaluated the Ox-PTM profile in cardiac and skeletal muscles of rats bred for low (LCR) and high (HCR) intrinsic aerobic capacity. Redox proteomics screening revealed different cysteine (Cys) Ox-PTM profile between HCR and LCR rats. HCR showed a higher number of oxidized Cys residues in skeletal muscle compared to LCR, while the opposite was observed in the heart. Most proteins with differentially oxidized Cys residues in the skeletal muscle are important regulators of oxidative metabolism. The most oxidized protein in the skeletal muscle of HCR rats was malate dehydrogenase (MDH1). HCR showed higher MDH1 activity compared to LCR in skeletal, but not cardiac muscle. These novel findings indicate a clear association between Cys Ox-PTMs and aerobic capacity, leading to novel insights into the role of Ox-PTMs as an essential signal to maintain metabolic homeostasis.

## Introduction

High aerobic capacity (*i*.*e*. the capacity to use oxygen), is associated with metabolic benefits, cardiovascular protection and longevity^1–5^. To determine the role of intrinsic aerobic capacity to prevent metabolic diseases and improve longevity, artificial selections for low and high intrinsic running capacity (LCR/HCR) were performed in rats^6,7^. Low-capacity runner (LCR) rats are insulin-resistant, hyperglycemic, hyperlipidemic, obese and present high cardiovascular risk factors compared to high-capacity runners (HCR) rats^7–9^. Remarkably, survival rate is ~45% higher in HCR than LCR rats^9^. While this rat model establishes that intrinsic components of oxidative energy metabolism are inherently associated with health and longevity, elucidating the mechanisms by which high aerobic capacity regulates whole-body metabolic homeostasis is a major research challenge.

Skeletal and cardiac muscles enhance the production of reactive oxygen species (ROS) during high-energy demands, such as during myofibers contraction^10^. ROS consists of radicals and non-radical species produced by the partial reduction of oxygen in different sources, including mitochondria, NADPH oxidase and peroxisomes^11^. ROS can induce reversible and irreversible modifications to different proteins. Examples of reversible modifications are disulfides, S-glutathionylation and S-nitrosylation, while examples of irreversible are carbonylation and sulfonic acid^12^. Cysteine (Cys) is an amino acid highly susceptible to reversible oxidative post-translational modifications (Ox-PTMs) due to the presence of a thiol side chain. Cys Ox-PTMs may regulate cellular homeostasis in several tissues, including skeletal and cardiac muscles^13–18^, but the putative relationship between Ox-PTM and intrinsic components of oxidative energy metabolism is poorly understood. In this sense, mass-spectrometry-based proteomic analysis is a powerful tool to investigate the post-translational modifications of the proteome, including Ox-PTMs^18^.

Here, we hypothesized that Ox-PTMs of cysteine are associated with high intrinsic aerobic capacity in skeletal and cardiac muscles. We determined the metabolic phenotype and the Cys Ox-PTM profile in both skeletal and cardiac muscles of LCR and HCR rats. HCR rats had superior mitochondrial content and total (GSH) and oxidized glutathione (GSSG) levels compared to LCR rats in skeletal muscle, while only modest changes were observed in cardiac muscle. A redox proteomic screening revealed a different Cys Ox-PTM profile between HCR and LCR. HCR rats showed higher number of oxidized Cys residues in proteins of skeletal muscle than LCR, while the opposite pattern was observed in the heart. The most significantly oxidized protein in the skeletal muscle of HCR rats was malate dehydrogenase (MDH1), and HCR rats showed higher MDH1 activity than LCR rats in the skeletal muscle, but not in the cardiac muscle. This study provides new insights into the role of Cys Ox-PTMs as essential signals to maintain metabolic homeostasis and opens the perspective to explore Ox-PTMs to counteract metabolic diseases.

## Results

### HCR rats display higher aerobic capacity, lower body mass and slower resting heart rate

**Figure 1:**
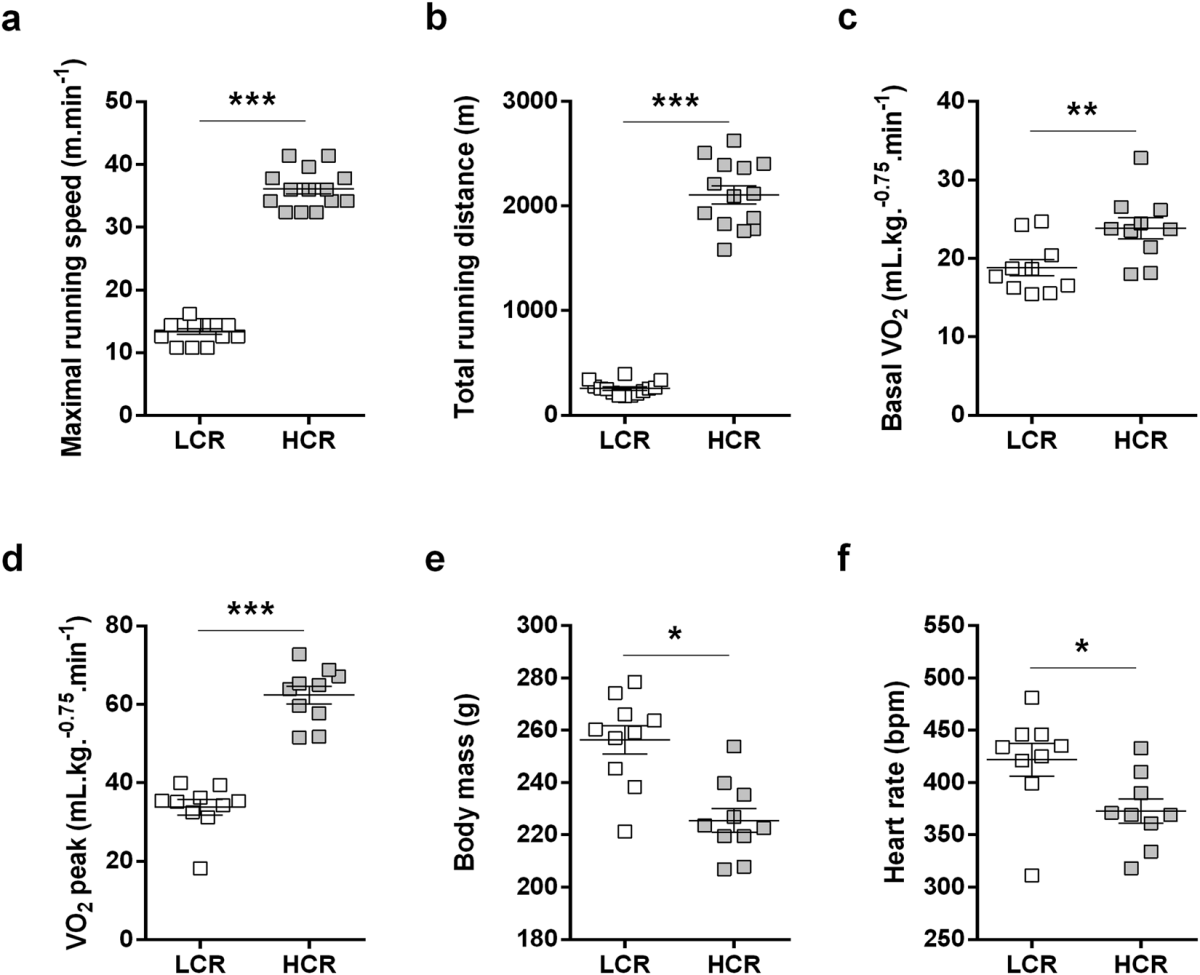
Aerobic capacity, body mass and basal heart rate in HCR and LCR rats. (**a**) Maximal running speed. (**b**) Total running distance. (**c**) Basal oxygen consumption (VO_2_). (**d**) Peak oxygen consumption (VO_2 peak_). (**e**) Body mass. (**f**) Basal heart rate. Data are presented as mean ± SEM with dots as individual values. *p < 0.05, **p < 0.01, ***p < 0.001 HCR as compared to LCR, n = 10.

To confirm whether the 32° generation of HCR/LCR rats maintains the distinctive phenotypes of high and low intrinsic aerobic capacity, we assessed oxygen consumption (VO_2_) during a maximal incremental running test. As expected, HCR rats displayed remarkable running performance (Fig. 1a,b), and higher basal VO_2_ and peak oxygen consumption (VO_2peak_) than LCR rats (Fig. 1c,d). HCR rats also displayed lower body weight (Fig. 1e) and slower resting heart rate compared to LCR rats (Fig. 1f). No differences were observed in *plantaris* muscle mass, heart mass and other basal and dobutamine stress echocardiographic parameters between HCR and LCR rats (Table 1 and Supplemental Table 1). These data confirm that the present cohort of HCR and LCR rats is an animal model of high and low intrinsic aerobic capacity.

**Table 1:** Plantaris muscle and cardiac muscle mass and echocardiography in LCR and HCR rats.

	LCR (n = 10)	HCR (n = 10)
*Plantaris* (g)	0.23 ± 0.01	0.20 ± 0.01
Left ventricle (g)	0.54 ± 0.02	0.55 ± 0.01
Right ventricle (g)	0.16 ± 0.01	0.14 ± 0.01
Atria (g)	0.04 ± 0.01	0.04 ± 0.01
Left ventricular anterior wall thickness in diastole (mm)	1.47 ± 0.16	1.37 ± 0.11
Left ventricular anterior wall thickness in systole (mm)	2.40 ± 0.14	2.29 ± 0.17
Left ventricular end-diastolic diameter (mm)	6.09 ± 0.22	6.04 ± 0.16
Left ventricular end-systolic diameter (mm)	3.14 ± 0.24	3.00 ± 0.22
Fractional shortening (%)	45.68 ± 1.88	47.06 ± 2.78
Ejection fraction (%)	75.33 ± 2.15	76.57 ± 2.81

Data are expressed as mean ± SEM.

### HCR rats have higher mitochondrial content and antioxidant capacity in the skeletal muscle

**Figure 2:**
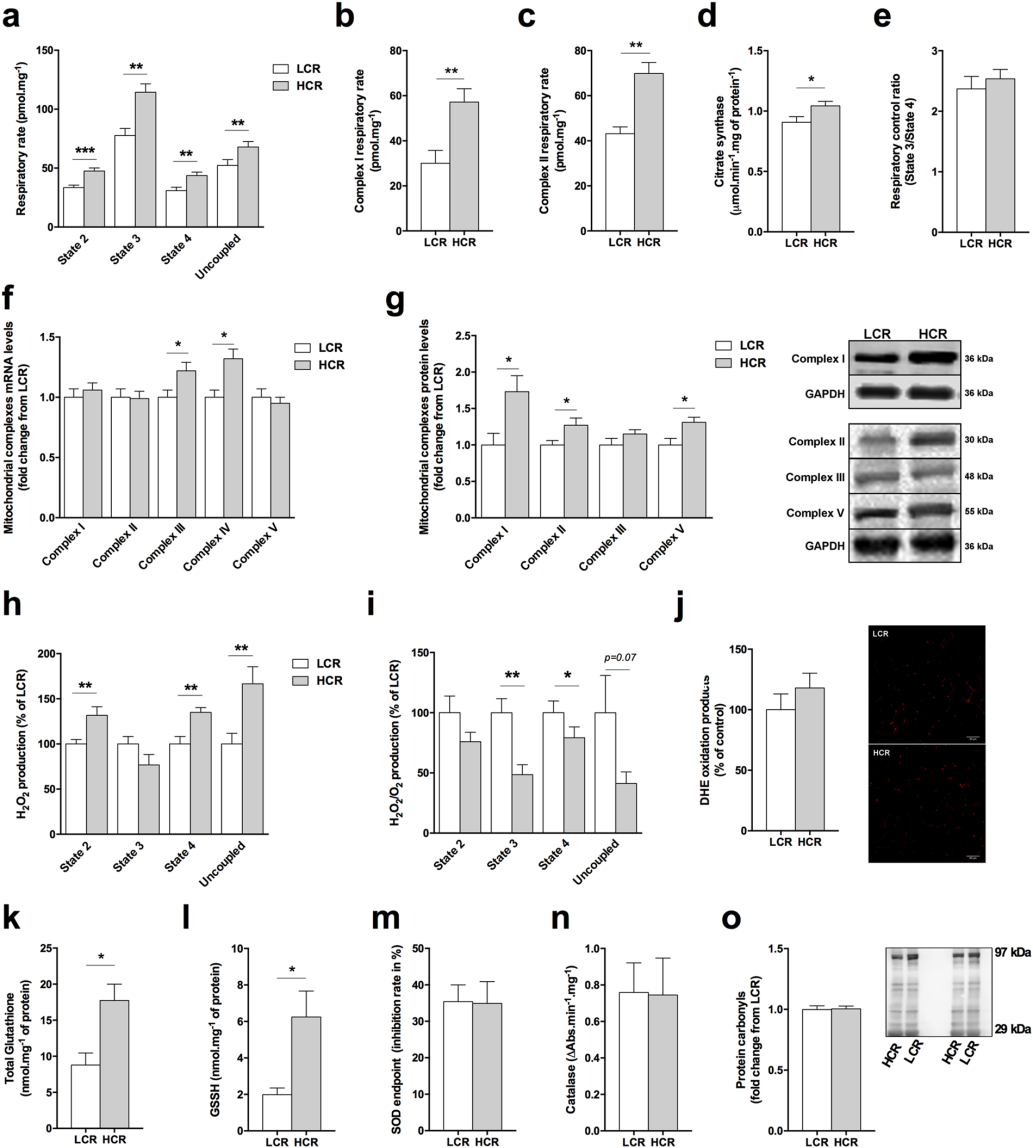
Skeletal muscle metabolic phenotype in HCR and LCR rats. (**a**) Oxygen consumption rate in *plantaris* muscle fibers. (**b**) Complex I and (**c**) complex II oxygen respiratory rate in *plantaris* muscle fibers. (**d**) Citrate synthase activity in *plantaris* muscle. (**e**) Respiratory control ratio (State 3/State 4) in *plantaris* muscle fibers. (**f**) Mitochondrial complexes mRNA expression in *plantaris* muscle. (**g**) Quantification of mitochondrial complexes and representative immunoblots in *plantaris* muscle. (**h**) Total H_2_O_2_ production and (**i**) H_2_O_2_ production normalized by oxygen respiratory rate in *plantaris* muscle fibers. (**j**) Quantification of fluorescent dihydroethidium (DHE) oxidation products and representative images in *plantaris* muscle cross-sections. (**k**) Total glutathione and (**l**) oxidized glutathione (GSSG) levels in *plantaris* muscle. (**m**) SOD and (**n**) catalase activity. (**o**) Quantification of carbonyl protein levels in *plantaris* muscle and representative immunoblots. Data are presented as mean ± SEM *p < 0.05, **p < 0.01, ***p < 0.001 HCR as compared to LCR, n = 6–12.

To determine whether HCR and LCR rats have a different skeletal muscle oxidative phenotype, we measured the mitochondrial respiratory rate in isolated *plantaris* muscle fibers. HCR displayed higher respiratory rate than LCR rats (Fig. 2a). Independent experiments in the presence of inhibitors for mitochondrial complexes I (rotenone) or II (malonic acid) revealed that both complexes have contributed to the higher state 3 respiratory rate observed in the HCR rats (Fig. 2b,c). HCR rats also presented higher citrate synthase activity than LCR rats in the *plantaris* muscle (Fig. 2d), while no difference was observed between groups neither in the respiratory control ratio (Fig. 2e) nor when respiratory rate data was normalized by citrate synthase activity (data not shown). Thus, our data suggest that mitochondrial respiratory rate is higher in HCR rats due to a higher mitochondrial content in the skeletal muscle. To test whether HCR rats display higher mitochondrial content than LCR, we evaluated the mRNA expression and protein content of the mitochondrial complexes, and we confirmed higher abundance of mitochondrial complexes in HCR than LCR (Fig. 2f,g and Supplemental Fig. 2).

Consistent with a higher mitochondrial content and respiratory rate, absolute hydrogen peroxide (H_2_O_2_) emission rate was increased in HCR rats compared with LCR rats in the *plantaris* muscle (Fig. 2h). However, HCR rats produced lower H_2_O_2_ when data were normalized to resting oxygen consumption (Fig. 2i). HCR and LCR rats also have similar fluorescent DHE oxidation products in *plantaris* muscle cross-sections (Fig. 2j). HCR rats displayed higher total glutathione levels and oxidized glutathione (GSSG) than LCR rats in *plantaris* muscle (Fig. 2k,l), but no changes were observed in superoxide dismutase (SOD) or catalase activity (Fig. 2m,n). In addition, HCR and LCR rats did not show differences in the protein carbonyl levels (Fig. 2o), which is an irreversible oxidation reaction and a biomarker of oxidative damage^19^. Therefore, these findings show that HCR’s skeletal muscles are better prepared than LCR’s to cope with oxidative damage.

### HCR rat’s metabolic phenotype in the cardiac muscle

**Figure 3:**
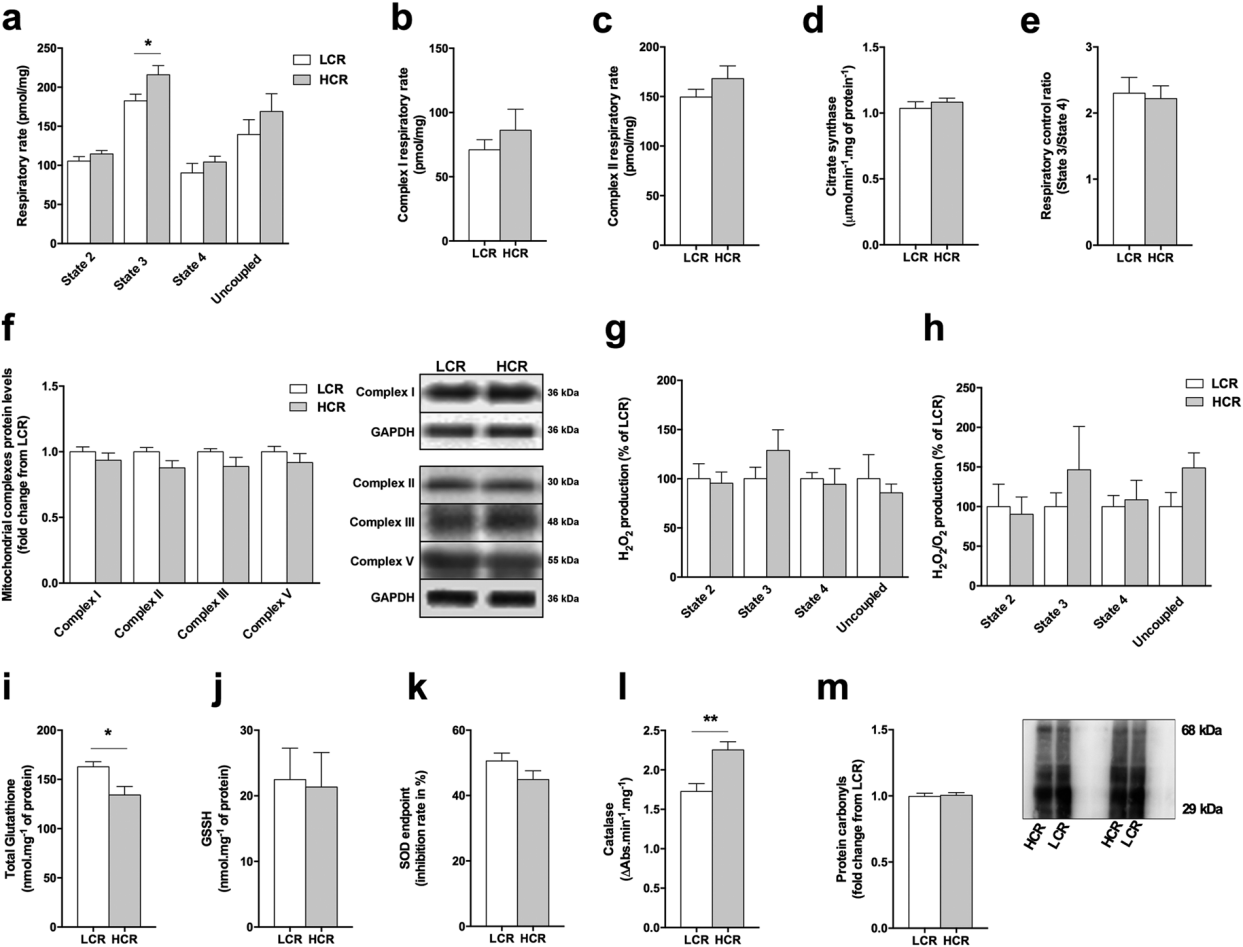
Heart metabolic phenotype in HCR and LCR rats. (**a**) Oxygen consumption rate in cardiac muscle fibers. (**b**) Complex I and (**c**) complex II oxygen respiratory rate in cardiac muscle fibers. (**d**) Citrate synthase activity in cardiac muscle. (**e**) Respiratory control ratio (State 3/State 4) in cardiac muscle fibers. (**f**) Quantification of mitochondrial complexes and representative immunoblots in cardiac muscle. (**g**) Total H_2_O_2_ production and (**h**) H_2_O_2_ production normalized by oxygen respiratory rate in cardiac muscle fibers. (**i**) Total glutathione and (**j**) oxidized glutathione (GSSG) levels in cardiac muscle. (**k**) SOD and (**l**) catalase maximal activity. (**m**) Quantification of carbonyl protein levels and representative immunoblots in cardiac muscle. Data are presented as mean ± SEM. *p < 0.05, **p < 0.01 HCR as compared to LCR, n = 6–12.

Because previous evidence suggested that metabolic and oxidative profiles can differ significantly between cardiac and skeletal muscles, we evaluated whether the differences observed in the skeletal muscle would display a similar or distinct pattern in the cardiac muscle. In this way, HCR displayed a modest increase in state 3 respiratory rate (Fig. 3a), with no differences in the respiratory rate when the cardiac muscle fibers were incubated with inhibitors for mitochondrial complexes I (Fig. 3b) or II (Fig. 3c). In addition, HCR and LCR rats displayed similar citrate synthase activity (Fig. 3d), respiratory control ratio (State 3/State 4) (Fig. 3e), mitochondrial complexes protein content (Fig. 3f), and H_2_O_2_ production (Fig. 3g,h). HCR rats presented lower total glutathione levels than LCR rats with similar GSSG levels (Fig. 3i,j) and SOD activity (Fig. 3k). In contrast, HCR rats displayed higher catalase activity than LCR rats (Fig. 3l) and unchanged protein carbonyl levels (Fig. 3m). These findings suggest that HCR rats have a modest increase in mitochondrial respiratory rate and antioxidant defense in the cardiac muscle when compared with LCR rats.

### HCR and LCR rats display different Ox-PTM profile between skeletal and cardiac muscles

The data described above demonstrate that HCR rats have a pronounced increase in oxidative metabolism in the skeletal muscle while a modest increase in the cardiac muscle. To determine whether Ox-PTMs are associated with the different muscle phenotypes observed in HCR and LCR rats, we screened redox regulated proteins in both the skeletal and cardiac muscles. We first performed fluorescent two-dimensional gel eletrophoresis (2D-GE) of *plantaris* muscle proteins to visualize and compare Cys redox state between HCR and LCR rats. *Plantaris* muscle Cys residues were labeled with fluorescent dyes absorbing and emitting at different wavelengths on the infrared region (DY-680 and DY-780, Dynomics) for reduced (red color-DY-780) and oxidized (green color-DY-680) thiol groups. After 2D-GE, the ratio of the intensity between the two fluorophores (oxidized:reduced Cys residues) at each spot reflects the Cys residues redox state of corresponding protein. As the value obtained is a ratio, it is independent of protein amount, allowing a more precise comparison of LCR and HCR cell extracts independently separated by 2D-GE. Of interest, infrared scanning of fluorescent gels revealed a more oxidized redox status of HCR *plantaris* proteins when compared with LCR (Supplemental Fig. 1).

**Figure 4:**
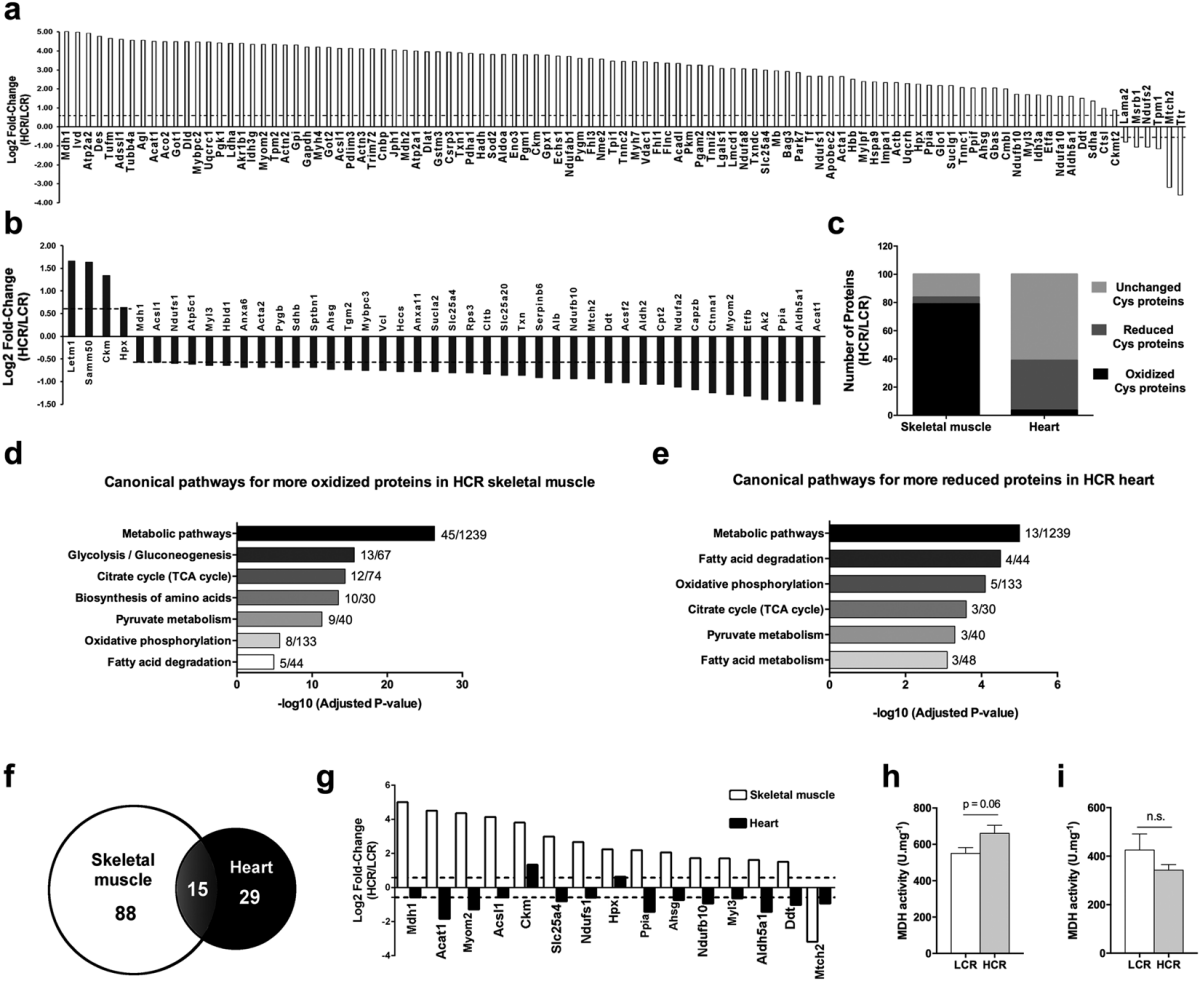
Oxidative post-translational modifications (Ox-PTMs) of cysteine in skeletal muscle and heart in HCR and LCR rats. (**a**,**b**) Differentially redox modulated proteins (p < 0.05 and ≥1.5-fold change) in (**a**) *plantaris* muscle and (**b**) heart. Data are presented as log2 fold-change of HCR/LCR ratio intensity. (**c**) Percentage of unchanged, reduced and oxidized proteins in *plantaris* muscle and heart. (**d**,**e**) Pathway enrichment analysis using redox modulated (reduced and oxidized) proteins from (**d**) *plantaris* muscle and (**e**) heart. (**f**) Venn diagram with redox modulated (reduced and oxidized) proteins in *plantaris* muscle and heart. (**g**) All 15 redox modulated proteins in both *plantaris* muscle and heart. (**h**,**i**) Malate dehydrogenase 1 (MDH1) activity in (**h**) *plantaris* and (**i**) cardiac muscles. MDH1 activity data are presented as mean ± SEM (n = 8 rats/group).

To circumvent the limitation of 2D-GE method, which fails to visualize less abundant proteins in our complex (tissue) samples, we used a gel-free proteomic analysis employing Cys-specific isotopic coded affinity tags (OxICAT), of skeletal and cardiac muscles from HCR and LCR rats. OxICAT revealed 123 proteins in all biological replicates of *plantaris* muscle, of which 107 displayed higher oxidized/reduced ratio (p < 0.05 and ≥1.5-fold change) in HCR than LCR, 10 were not different and only 6 proteins with lower oxidized/reduced ratio in HCR than LCR (Fig. 4a and Table Supplemental 2). The top 20 redox-modulated proteins in *plantaris* muscle are presented in Table 2. OxICAT also revealed 113 proteins in cardiac muscle. From these 113 proteins, 4 proteins had higher oxidized/reduced ratio (p < 0.05 and ≥1.5-fold change), 67 proteins had unchanged oxidized/reduced ratio, and 42 proteins had lower oxidized/reduced ratio in HCR when compared to LCR (Fig. 4b and Table Supplemental 3). The top 20 redox-modulated proteins in the cardiac muscle are presented in Table 3. Taken together, these data demonstrate that HCR rats presented higher levels of oxidized proteins than LCR rats in the skeletal muscle, while the opposite was observed in cardiac muscle, since more reduced proteins were observed in HCR hearts (Fig. 4c).

**Table 2:** Top 20 redox modulated proteins in the *plantaris* muscle.

Protein	ICAT-modified Cysteine	Fold change HCR/LCR
Malate dehydrogenase, cytoplasmic (Mdh1)	Cys137/154	5.01
Isovaleryl-CoA dehydrogenase, mitochondrial (Ivd)	Cys353	4.99
Sarcoplasmic/endoplasmic reticulum calcium ATPase 2 (Atp2a2)	Cys471/560/680	4.93
Desmin (Des)	Cys337	4.77
Elongation factor Tu, mitochondrial (Tufm)	Cys129/294	4.66
Adenylosuccinate synthetase isozyme 1 (Adssl1)	Cys64/186	4.62
Protein Tubb4a (Tubb4a)	Cys12/308	4.56
Amylo-1, 6-glucosidase, 4-alpha-glucanotransferase isoform CRA_a (Agl)	Cys82/128/174/287/779 Cys972/1065/1277	4.56
Acetyl-CoA acetyltransferase, mitochondrial (Acat1)	Cys196	4.51
Aconitate hydratase, mitochondrial (Aco2)	Cys128/391/416/752	4.5
Aspartate aminotransferase, cytoplasmic (Got1)	Cys84/397	4.49
Dihydrolipoyl dehydrogenase, mitochondrial (Dld)	Cys70/492	4.49
Myosin binding protein C, fast-type (Mybpc2)	Cys159/335/352/447/479/566 Cys622/819/1019/1041	4.48
Cytochrome b-c1 complex subunit 1, mitochondrial (Uqcrc1)	Cys272/386/416	4.47
Phosphoglycerate kinase 1 (Pgk1)	Cys50/321	4.43
Methionine-R-sulfoxide reductase B1 (Msrb1)	Cys26	−1.08
NADH dehydrogenase iron-sulfur protein 2, mitochondrial (Ndufs2)	Cys352	−1.08
Tropomyosin alpha-1 chain (Tpm1)	Cys193	−1.15
Mitochondrial carrier homolog 2 (C. elegans) (Mtch2)	Cys80/99	−3.19
Transthyretin (Ttr)	Cys30	−3.6

Table includes the redox ratio of individual Cys residues within those proteins. The redox state of selected redox Cys residues labeled with both light ^12^C-form and heavy ^13^C-form was calculated using the oxidation ratio (oxidized:reduced).

**Table 3:** Top 20 redox modulated proteins in the heart.

Protein	ICAT-modified Cysteine	Fold Change HCR/LCR
LETM1 and EF-hand domain-containing protein 1, mitochondrial (Letm1)	Cys560	1.66
Sorting and assembly machinery component 50 homolog (Samm50)	Cys66	1.64
Creatine kinase M-type (Ckm)	Cys148/258	1.34
Hemopexin (Hpx)	Cys150/233/259/412	0.64
Serum albumin (Alb)	Cys58/127/204/293/307/ 317/391/422/469/519/600	−0.94
Protein Ndufb10 (Ndufb10)	Cys78/127	−0.94
Mitochondrial carrier homolog 2 (C. elegans) (Mtch2)	Cys80/99	−0.94
D-dopachrome decarboxylase (Ddt)	Cys24	−1.03
Acyl-CoA synthetase family member 2, mitochondrial (Acsf2)	Cys78/469	−1.03
Aldehyde dehydrogenase, mitochondrial (Aldh2)	Cys69/394	−1.06
Carnitine O-palmitoyltransferase 2, mitochondrial (Cpt2)	Cys85/653	−1.06
NADH dehydrogenase [ubiquinone] 1 alpha subcomplex subunit 2 (Ndufa2)	Cys22/56	−1.12
F-actin-capping protein subunit beta (Capzb)	Cys63	−1.18
Catenin (Cadherin associated protein), alpha 1 (Ctnna1)	Cys118/344/536	−1.25
Myomesin 2 (Myom2)	Cys198/889/1203/1252/ 1272	−1.29
Electron transfer flavoprotein subunit beta (Etfb)	Cys42	−1.32
Isoform 2 of Adenylate kinase 2, mitochondrial (Ak2)	Cys93	−1.4
Peptidyl-prolyl cis-trans isomerase A (Ppia)	Cys21/163	−1.43
Succinate-semialdehyde dehydrogenase, mitochondrial (Aldh5a1)	Cys82/333	−1.43
Acetyl-CoA acetyltransferase, mitochondrial (Acat1)	Cys117/416	−1.84

Table includes the redox ratio of individual Cys residues within those proteins. The redox state of selected redox Cys residues labeled with both light ^12^C-form and heavy ^13^C-form was calculated using the oxidation ratio (oxidized:reduced).

We also performed pathway enrichment analysis using KEGG^20^, a database resource for understanding high-level functions of biological system, which has well-annotated metabolic pathways. KEGG analysis revealed that metabolic pathways were highly enriched by Ox-PTMs in both the skeletal (Fig. 4d and Table Supplemental 4) and cardiac (Fig. 4e and Table Supplemental 5) muscles. Of note, there were overlaps between pathways identified in the skeletal and cardiac muscle (Fig. 4d,e). We next cross-referenced the lists of redox modulated residues and found 15 proteins present in both lists (Fig. 4f). Twelve of these proteins had more oxidized levels in the skeletal muscle, but more reduced levels in the cardiac muscle of HCR rats (Fig. 4g). The exceptions were the Creatine Kinase M-type (CKM), and Hemopexin (HPX) that displayed more oxidized levels in both the skeletal and cardiac muscles of HCR (Fig. 4g) and the Mitochondrial Carrier Homolog 2 (MTCH2) that presented reduced levels in both tissues of HCR rats.

### HCR rats show higher malate dehydrogenase (MDH1) activity than LCR rats in the skeletal muscle, but not in the cardiac muscle

Our data indicate that HCR rats have more oxidized proteins in the skeletal muscle and many of these oxidized proteins are important regulators of the oxidative metabolism, including some essential metabolic enzymes. To determine whether there was an association between the redox state and the activity of metabolic enzymes, we measured the activity of the MDH1, the most significantly oxidized protein in the skeletal muscle of HCR rats. Interestingly, HCR rats display a trend toward increased MDH1 activity (p = 0.06) in the skeletal muscle when compared with LCR rats (Fig. 4h), while no significant differences were observed in cardiac muscle MDH1 activity (Fig. 4i). Thus, contrary to what is generally observed for many oxidized enzymes, increased levels of Ox-PTMs in MDH1 did not mediate any loss of catalytic activity. Rather, a slight but sub-significantly increase in activity was observed in *plantaris* muscle of HCR rats, a muscle type that displays higher mitochondrial content and antioxidant capacity in HCR than LCR rats.

## Discussion

Given the association between high aerobic capacity and the prevention of metabolic diseases^4^, elucidating the mechanisms by which high aerobic capacity regulates whole-body metabolic homeostasis is a major research challenge. To test the hypothesis that Ox-PTMs would be associated with intrinsic oxidative capacity in muscle, we applied redox proteomic approach in skeletal and cardiac muscles of a rat model of low and high intrinsic aerobic capacity. We found a clear association between Ox-PTMs and intrinsic aerobic capacity in both the skeletal and cardiac muscles. HCR rats displayed a higher ratio of oxidized to reduced Cys residues in *plantaris* muscle than LCR rats whereas the opposite was observed for cardiac muscle with a lower ratio of oxidized to reduced Cys residues than LCR rats. Many of these redox-modulated proteins are important regulators of the oxidative metabolism, including the metabolic enzyme MDH1. Notably, HCR rats show increased MDH1 activity in the skeletal muscle, but not in the heart. These findings open the perspective to consider Ox-PTMs as targets to regulate metabolic homeostasis in the skeletal and cardiac muscle.

In the current study, we used a well-established rat model of high/low intrinsic aerobic capacity, which recapitulates many of the metabolic differences observed in humans. This model originated from a heterogeneous founder population with breeder selection based on the intrinsic running capacity of each rat. The selection started in 1996 and a rotational breeding was performed at each generation^6,21^. The 32° generation HCR/LCR rats used in this study kept the phenotype observed in previous generations^7,8,21–24^ showing 8.2-fold increase in the maximal running distance between HCR and LCR rats, which is superior to the data reported in other generations^7,23^, and similar to the observed in the 36° generation^21^. The increased maximal running distance and peak oxygen uptake were associated with improved mitochondrial function and redox homeostasis in the skeletal muscle (Fig. 2), but not with cardiac function (Table 2) or cardiac mitochondrial function (Fig. 3).

HCR rats showed higher resting VO_2_ and lower resting heart rate than LCR rats. The lower resting heart rate (i.e. bradycardia) has been used as an index of improved aerobic capacity and has been associated with improved longevity^25^. Yet, the increased resting VO_2_ in HCR rats may be explained by increased energy dissipation in skeletal muscle as heat, once the higher heat production might be due to increased mitochondrial density observed in the skeletal muscle of HCR rats^26^. A well-known protein related to heat production is uncoupling protein (UCP). In skeletal muscle, UCP3 may play a major role in energy expenditure and also participate in the determination of mitochondrial efficiency^27^. In fact, high UCP3 expression has been observed in skeletal muscle of HCR rats^28^. Ox-PTM can either activate or signal for increases in UCP expression without decreasing oxidative phosphorylation efficiency^29,30^. Indeed, the increased skeletal muscle mitochondrial uncoupling could reduce the oxidative damage in HCR rats, which was observed in our study when we normalized H_2_O_2_ production per O_2_ consumption. In this sense, the greater number of oxidized proteins in *plantaris* muscle of HCR animals could be influencing the mitochondrial uncoupling to reduce the disruption of redox signaling and control damage, resulting in an adaptive role of reactive species as essential signaling molecules in HCR rats.

Although absolute H_2_O_2_ emission rate is higher in HCR’s than LCR’s plantaris muscle, HCR muscle fibers may also possess a more efficient antioxidant system than LCR, so that for each oxygen molecule consumed, less H_2_O_2_ accumulates in HCR cells. These differences may not be explained by differential catalase activity, which was similar between HCR vs. LCR, but through other several key antioxidant enzymes (e.g. GSTM2, PDIA3 and HSPB7) increased in HCR when compared with LCR^31^.

We performed gel-free redox proteomics analysis in skeletal and cardiac muscles of HCR and LCR rats to identify and quantify reduced and oxidized states of the Cys residues. In our study, approximately 80% of the proteins identified in the redox proteomics of HCR *plantaris* protein extracts displayed a higher ratio of oxidized to reduced Cys residues, which was somewhat surprising. Considering that we did not find signs of oxidative damage in the skeletal muscle of HCR rats, we speculate that Ox-PTMs of Cys would not be deleterious for oxidative metabolism, especially because many of the oxidized proteins in HCR rats are enzymes involved in the intermediary metabolism. Ox-PTM of Cys residues are thought to reduce enzyme activities, however the type and degree of the oxidation depends on many factors, such as the pH of the microenvironment, surrounding amino acids and whether the oxidation is mediated enzymatically or non-enzymatically^32^. Accumulating evidence suggests that Ox-PTMs of Cys have functional effects by altering the activity of the proteins even when the protein content has not changed^33,34^. Based on that, we evaluated the MDH1 activity in *plantaris* muscle of HCR and LCR rats, the enzyme with highest ratio of oxidized to reduced Cys residues in HCR among all redox modulated proteins in the skeletal muscle. MDH1 is known for catalyzing the reversible oxidation of malate to oxaloacetate in many metabolic pathways, including the Krebs cycle and malate/aspartate shuttle. We found that Ox-PTM in the Cys137/154 of MDH1 could be associated with slightly increased MDH1 activity in HCR *plantaris* muscle.

The higher MDH1 activity could be associated with the presence of NADH in the Cys residues. Indeed, NADH being at or near to the enzymatic active center can prevent chemical modifications in dehydrogenases enzymes^35^. The Cys Ox-PTM identified in the *plantaris* muscle of HCR rats are near to the active and NAD binding sites, suggesting that the MDH1 oxidation in the Cys residues could be protected by the NADH, and not affected the enzyme activity. Another explanation comes from a recent study that reported that H_2_O_2_ affects kinetics, structure, and thermodynamic stability of MDH1 through Cys oxidation. Interestingly, others have observed that the thioredoxin-reversible homodimerization of MDH1 protects the protein from over oxidation, maintaining the MDH1 function^36^. These data suggest that a balance between oxidation and antioxidant system is necessary to maintain MDH1 and mitochondrial function during increased energy demand conditions.

**Figure 5:**
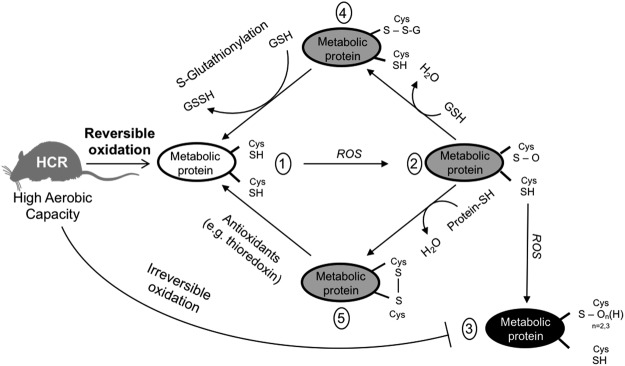
Proposed model for how reversible oxidative post-translational modifications (Ox-PTMs) may protect skeletal muscle from oxidative damage. Reactive cysteine thiols from a metabolic protein in HCR rats is shown with two exposed thiols (1). Oxidation by ROS can generate a sulfenic acid (2), which can be converted to irreversible sulfinic and sulfonic acid species (3). Instead, sulfonic acid can be converted to reversible forms, such as disulfides with protein (4) or disulfide with glutathione (5). Thus, by stimulating reversible oxidation processes (4 and 5), HCR rats prevents irreversible oxidation (3) in the skeletal muscle. Rat illustration was adapted from Servier Medical Art (smart.servier.com).

Cysteine residues on protein surfaces may act as redox buffers to protect proteins from irreversible oxidant damage^33,34,37^. Taking into consideration that: 1) HCR rats have higher number of oxidized proteins in the skeletal muscle and higher number of reduced proteins in the cardiac muscle than LCR rats, and 2) neither skeletal nor cardiac muscle of HCR show higher ROS production than LCR, it’s reasonable to assume that skeletal and cardiac muscles antioxidant defense is differentially regulated. In fact, HCR rats displayed higher GSH and GSSG content in the skeletal muscle, but not in the cardiac muscle, which was consistent with differences observed in the mitochondrial phenotype between these tissues. Reversible S-glutathionylation is an Ox-PTM in which a glutathione is added to Cys residues of a target protein. Based on our data, we speculate that an enhanced S-glutathionylation process in the skeletal muscle of HCR rats might protect several proteins from irreversible oxidation, preserving or even increasing the activity of these proteins, such as the MDH1. Overall, we propose that by stimulating reversible oxidation processes, HCR rats are preventing irreversible oxidation in the skeletal muscle (Fig. 5).

We acknowledge limitations to our study. First, the association between Ox-PTMs and improved oxidative metabolism indicates that Ox-PTMs may play a role in maintaining metabolic homeostasis. However, further studies exploring a causal relationship between Ox-PTMs of Cys residues and aerobic capacity are necessary. Second, we used a model of high vs. low intrinsic aerobic capacity, but we cannot extend these findings to other conditions of impaired aerobic capacity, such as type II diabetes and obesity, or enhanced aerobic capacity, resulting from endurance exercise training. We encourage new studies to evaluate the effects of metabolic diseases and/or exercise training in the skeletal and cardiac muscle Ox-PTM profile.

In summary, this study demonstrated that Ox-PTMs of Cys are associated with high intrinsic aerobic capacity, and higher ratio of oxidized to reduced Cys residues is positively associated with higher aerobic capacity in the skeletal muscle. This leads to new insights into the role of Ox-PTMs as a key process to maintain metabolic homeostasis under high-energy demand conditions and opens the perspective to explore Ox-PTMs to counteract metabolic diseases.

## Methods

### Animals and experimental design

All animal procedures were approved by the Norwegian Council for Animal Research (ID 5243), which was in accordance with the Guide for the Care and Use of Laboratory Animals by the European Commission Directive 86/609/EEC. The selection of rats yielding HCR and LCR has been previously described - for details see^6,7^. In the current study, two sets (20 animals in each set) of 12 weeks old male rats from the 32° generation were used. Animals were housed (four per cage) in an animal facility under controlled temperature (22 °C) with 12:12 hours light:dark cycle. Animals had *ad libitum* access to standard laboratory chow and water. The first set of HCR (n = 10) and LCR (n = 10) rats was weighed and submitted to a maximal incremental running test. One week after the running test, rats were anesthetized with isoflurane and killed by decapitation. The heart (atria and ventricles) and *plantaris* muscle were carefully harvested, weighed, snap-frozen and stored in −80 °C for further enzymatic and colorimetric assays, DHE fluorescent staining, immunobloting, real-time quantitative PCR (RT-qPCR) and redox proteomics. The second set of HCR (n = 10) and LCR (n = 10) rats was submitted to echocardiography. Rats were then anesthetized with isoflurane and killed by decapitation. Left ventricle and *plantaris* skeletal muscle were carefully harvested and immediately stored at 1) ice-cold biopsy preservation solution (BIOPS) for mitochondrial respiration and H_2_O_2_ emission measurements or 2) −80 °C for further enzymatic assays and redox proteomics experiments.

### Running capacity and oxygen uptake measurements

Rats were submitted to a maximal incremental running test in a metabolic chamber system. Each animal was adapted in treadmill exercise for three consecutive days (*i*.*e*. ~10 min in each day at running speed of 6 to 9 m.min^−1^). Rats were then submitted to a 15-min resting time in order to measure the resting oxygen uptake (VO_2_). After the resting measurement, animals ran on a treadmill at 15° inclination. The speed started at 6 m.min^−1^ and was increased by 2 m.min^−1^ every 2 min until rats were unable to run. The peak oxygen uptake (VO_2peak_), speed and distance were recorded.

### Echocardiography

Heart structure and function were performed using an echocardiography (Vevo 770, Visual Sonics, Toronto, Canada) during isoflurane (2%) anesthesia. Left ventricular anterior wall thickness in systole and diastole (LVAWs and LVAWd) were assessed. Left ventricular end-diastolic diameter and end-systolic diameter (LVEDD and LVESD) were used in order to calculate the fractional shortening (FS) as following: FC (%) = [(LVEDD - LVESD)/LVEDD] x 100. After baseline measurement, three different intravenous dobutamine infusions (5, 10 and 20 ug.kg-1.min-1) were performed and the same parameters were recorded after each dose administration^38^. All myocardial structures were manually measured according to the leading-edge method of the American Society of Echocardiography^39^. The examiner (NR) was blinded to the group’s allocation.

### Mitochondrial respiration and H_2_O_2_ emission measurements

Fiber bundles of cardiac and *plantaris* muscle were isolated and permeabilized by incubation in saponin (50 µg.ml^−1^). Fiber bundles were rinsed in ice-cold mitochondrial respiration medium containing 0.5 mM EGTA, 3 mM MgCl_2_, 60 mM K-lactobionate, 20 mM taurine, 10 mM KH_2_PO_4_, 20 mM Hepes, 110 mM sucrose and 1 g/l BSA essentially fatty acid free adjusted to pH 7.1^40^. High-resolution O_2_ consumption measurements and H_2_O_2_ emission were conducted in the OROBOROS O2K Oxygraph (OROBOROS INSTRUMENTS, Innsbruck, Austria). Chambers containing 2 ml of mitochondrial respiration medium at 37 °C was maintained with O_2_ levels between 400 and 600 µM. Malate (2 mM), glutamate (10 mM), succinate (10 mM), 10-acetyl-3,7-dihydroxyphenoxazine (Amplex Red; 25uM) and Horseradish peroxidase (HRP; 0,5 U.ml^−1^) were added in each chamber. Permeabilized fibers from cardiac (~2 mg) and *plantaris* skeletal muscle (~4 mg) were then added in the OROBOROS machines. State 2 O_2_ consumption rate was recorded. State 3 O_2_ consumption rate and H_2_O_2_ emission was recorded after stimulation with adenosine diphosphate (ADP; 1 mM). State 4 O_2_ consumption rate was recorded after inhibition of (ATP) synthase by oligomycin (2ug.ml^−1^). Finally, consecutive carbonilcyanide p-triflouromethoxyphenylhydrazone (FCCP) additions (0.5 mM) were performed until observe a *plateau* effect (*i*.*e*. uncoupler effect). In independent experiments, inhibitors for complex I (*i*.*e*. rotenone) and II (*i*.*e*. malonic acid) were added after ADP phase as an attempt to estimate how complex I and II inhibition affect O_2_ consumption rate and H_2_O_2_ emission, respectively. All analyses were performed in duplicate. Respiratory control ratio was calculated by dividing State 3 by State 4 O_2_ consumption rates, which demonstrates the tightness of the coupling between mitochondrial respiration and phosphorylation.

### Citrate synthase and malate dehydrogenase activities

Citrate synthase activity was determined using the Citrate Synthase Assay Kit (CS 0720, Sigma-Aldrich, USA). Malate dehydrogenase (MDA) activity was determined by a colorimetric method based on the reaction: oxaloacetate + NADH + H^+^ $\to$ L-malate + NAD^+^, in which the change in optical density at 340 nm per unit time is a measure of the malate dehydrogenase activity.

### Protein carbonyls

Protein carbonyls were assessed by using the OxyBlot Protein Detection Kit (S7150; Millipore, USA). Soluble proteins (20 µg) were denatured by SDS and derivatized by DNPH. Proteins were submitted to electrophoresis and immunoblotting accordingly the manufacturer’s instructions.

### Glutathione redox status

Total, reduced, and oxidized (GSSG) glutathione levels were measured by using the Glutathione Fluorescent Detection Kit (K006-F5; Arbor Assay, USA). In order to block the reduced GSSG, 2-Vinylpyridine (2VP) was used accordingly the manufacturer’s instructions.

### Superoxide dismutase activity

Superoxide dismutase (SOD) activity was assessed by using the Sigma 19160 SOD determination kit (Sigma-Aldrich, Switzerland).

### Catalase activity

Hydrogen peroxide (H_2_O_2_) decomposition was assessed by following the decay in sample absorbance at 240 nm in the presence of 10 mM H_2_O_2_^41^.

### DHE fluorescence staining

*Plantaris* cross-sections (10 μm) were incubated with DHE (5 μM) in a light-protected incubator at 37 °C for 30 min. The sections were washed with phosphate buffered saline and fluorescence was assessed by confocal microscopy as previously described^42^. Quantitative analysis of fluorescent images was performed with ImageJ (NIH, USA).

### Sample preparation for proteomics

*Plantaris* and cardiac tissue extracts were pooled and homogenized in degassed 2D buffer (7 M Urea, 2 M Thiourea, 2.5% CHAPS) without DTT (dithiothreitol) at pH 5.0. 300 μg of cardiac protein and 600 μg of *plantaris* protein were precipitated in methanol (4 V), chloroform (1 V) and H_2_O (3 V) and frozen for further labeling to proceed with gel-free and gel-based-proteomics.

### Two-dimensional difference gel electrophoresis (2D-DIGE)

2D-DE was performed for preliminary proteomic profiling of skeletal muscle in HCR and LCR rats. Skeletal muscle homogenates were submitted to a differential labeling of reduced (DY-680) and oxidized (DY-780) thiol group using two 2DE-compatible fluorescent dyes absorbing and emitting at different wavelengths of the infrared region^17^. Differences in Cys residues oxidation between HCR and LCR samples were quantified by the intensity of each fluorophore at each spot as previously described^18^. Spots intensity was quantified and compared between groups using the DECODON software (Greifswald, Germany). The results were confirmed in biological replicates.

### Oxidative isotope-coded affinity tags (OxICAT)

OxICAT was performed as previously described^13^. This method detects all reversibly oxidized Cys, whereas irreversible oxidation states are not detected. The ICAT reagent exists in an isotopically light ^12^C-form (*i*.*e*. light ICAT) and heavy ^13^C-form (*i*.*e*. heavy ICAT). Briefly, proteins were denatured in order to access and label all reduced Cys irreversibly with light ICAT. Proteins were then reduced using a thiol reductant Tris (2-carboxyethyl) phosphine and labeled with heavy ICAT. Therefore, this method generates chemically identical proteins, but with different mass of their ICAT-label accordantly to the redox state (light ICAT for the reduced proteins and heavy ICAT for the oxidized proteins). Each sample was digested with trypsin, purified and analyzed by mass spectrometry. Biological triplicate was performed using three different pool of samples for each group (n = 10, 5 and 5, respectively) for *plantaris* and cardiac muscle. Of note, OxICAT is a method able to detect only reversible oxidations.

### Mass spectrometry (MS) analysis

After desalting, peptides were dried down in a SpeedVac centrifuge and resuspended in 0.1% formic acid. Peptides were analyzed on a LC-MS/MS platform consisting of an Easy-nLC 1000 UHPLC system (Thermo Fisher Scientific Inc, USA) interfaced with an LTQ-Orbitrap Elite hybrid mass spectrometer (Thermo Fisher Scientific Inc, USA) via a nanospray ESI ion source (Proxeon, Odense). Peptides were injected into a C-18 trap column (Acclaim PepMap100, 75 μm i. d. x 2 cm, C18, 5 μm, 100 Å, Thermo Scientific, USA) and further separated on a C-18 analytical column (Acclaim PepMap100, 75 μm i. d. x 50 cm, C18, 3 μm, 100 Å, Thermo Scientific, USA) using a multistep gradient with buffer A (0.1% formic acid) and buffer B (CH3CN, 0.1% formic acid): From 0–6% B in 5 min, 6–20% B in 78 min, 20–25% B in 8 min, 25–40% B in 5 min, 40–100% B in 4 min, 100% B in 10 min, 100–0% B in 1 min and 9 min with 100% A. The flow rate was 250 nl/min. Peptides eluted were analyzed on the LTQ-Orbitrap Elite hybrid mass spectrometer operating in positive ion- and data dependent acquisition mode using the following parameters: Electrospray voltage 1.9 kV, CID fragmentation with normalized collision energy 35, automatic gain control target value of 1E6 for Orbitrap MS and 1E3 for MS/MS scans. Each MS scan (m/z 400–1600) was acquired at a resolution of 120,000 FWHM, followed by 20 MS/MS scans triggered for intensities above 500, at a maximum ion injection time of 200 ms for MS and 120 ms for MS/MS scans.

### Protein identification, quantitation, and analysis

Raw data files were analyzed in Proteome Discoverer 1.4 (Thermo Fisher Scientific Inc., US) using the SEQUEST HT search engine with the Dec 2013 version of the Rat protein sequence database from UniProt (UniProt Consortium). Enzyme specified as trypsin with maximum of two missed cleavages allowed was searched. Precursor mass tolerance was 10 ppm and fragment mass tolerance was 0.6 Da. The Cys residue (labeled with OxICAT) oxidation was set as dynamic modification. The Percolator tool was used for peptide validation and a cutoff value of 0.01 for false discovery rate. Only peptides with high confidence were used for final protein identification. In order to further understand the biological relevance of the identified proteins, we performed functional enrichment analysis in the context of the Kyoto Encyclopedia of Genes and Genomes (KEGG) databases using the Enrichr^43^ and QIAGEN’S Ingenuity Pathway Analysis (IPA). A p value cut-off of 0.001 was used to identify enriched processes.

### Real-time quantitative PCR (RT-qPCR)

RNA transcript levels from *plantaris* muscle were assessed by RT-qPCR, as described before^44^. Total RNA was isolated using RNeasy Mini kit (Qiagen, USA) and RNA concentration and integrity were assessed. cDNA was synthesized using High Capacity cDNA Reverse Transcription Kit (Life Technologies, USA), and the genes NADH-ubiquinone oxidoreductase subunit A9 (Ndufa9), succinate dehydrogenase complex subunit B (Sdhb), ubiquinol-cytochrome c reductase core protein II (Uqcrc2), cytochrome c oxidase subunit I (Cox1), and ATP synthase H + transporting mitochondrial F1 complex alpha subunit 1 (Atp5a1) were analyzed. After testing three control genes, β-actin (Actb), cyclophilin, and hypoxanthine phosphoribosyl transferase 1 (Hprt1), we used Hprt1 as reference gene to normalize the data as it recorded the highest stability. Primers were designed using Primer-BLAST (NCBI) and primer sequences are listed in Table 4.

**Table 4:** Primer sequences for RT-qPCR mRNA analysis.

Target mRNA	PCR Primer sequence 5′ → 3′	Product Size (bp)	GenBank Accession #
Ndufa9	F: CTTCCAATGTCACGTCCTGC	105	NM_001100752.1
	R: GCCACCTTTCCCATGAGGTAT		
Sdhb	F: ATGCAGAGAAGGGATCTGTGG	90	NM_001100539.1
	R: CCAAGGTCTGTGTCGATCCT		
Uqcrc2	F: CCTCAAAGTTGCCCCAAAGC	77	NM_001006970.1
	R: TGGTAAACTCAAGTTCCTGAGGC		
Cox4i1	F: GCAGCAGTGGCAGAATGTTG	80	NM_017202.1
	R: CCGAAGGCACACCGAAGTAG		
Atp5a1	F: TGAACTGTTGGGCCGTGTAG	67	NM_023093.1
	R: GGAACCAACTGGACCCTTCC		
Actb	F: CCTTCTTGGGTATGGAATCCTGT	86	NM_031144.3
	R: GAGGTCTTTACGGATGTCAACG		
Cyclophilin	F: TGGCAAGCATGTGGTCTTTGGGAAG	103	NM_017101.1
	R: GGTGATCTTCTTGCTGGTCTTGCCATTC		
Hprt1	F: CAGTCCCAGCGTCGTGATT	138	NM_012583.2
	R: GCAAGTCTTTCAGTCCTGTCCAT		

### Imunoblotting

Protein isolation, immunoblotting, and detection were performed as previously described^42,45^. Immunoblots were incubated with antibodies against oxidative phosphorylation complexes (OXPHOS, MitoSciences/Abcam, #MS604), NDUFA9 (Abcam, #14713), and GAPDH (Santa Cruz, #sc-20358).

### Statistical analysis

Data are presented as mean ± standard error mean (SEM). Experimental groups were compared using unpaired two-tailed Student’s *t*-test. Statistical significance was set as p < 0.05.

## Electronic supplementary material

Supplementary Files
