# Supplementary material for: Differential regulation of cysteine oxidative post-translational modifications in high and low aerobic capacity
Source: Sci Rep. 2018 Dec 11;8:17772. doi: 10.1038/s41598-018-35728-2 (PMC6289973; doi:10.1038/s41598-018-35728-2)
Supplement: Supplementary file 1 — Supplementary Files [file 41598_2018_35728_MOESM1_ESM.pdf]

Supplementary Files

Differential regulation of muscle cysteine oxidative post-translational modifications in high and low aerobic capacity

Rodrigo W. A. Souza[1], Christiano R. R. Alves[1,2], Alessandra Medeiros[3], Natale Rolim[4], Gustavo J. J. Silva[4], José B. N. Moreira[4], Marcia N. Alves[4], Martin Wohlwend[4], Mohammed Gebriel[5], Lars Hagen[5], Animesh Sharma[5], Lauren G. Koch[6], Steven L. Britton[7,8], Geir Slupphaug[5], Ulrik Wisløff[4,9], Patricia C. Brum[1]*

[1]School of Physical Education and Sport, University of São Paulo, São Paulo, Brazil

[2]Section on Integrative Physiology and Metabolism, Joslin Diabetes Center, Harvard Medical School, Boston, Massachusetts, USA

[3]Biosciences Department, Federal University of São Paulo, Santos, Brazil

[4]K.G. Jebsen Center of Exercise in Medicine, Department of Circulation and Medical Imaging, Norwegian University of Science and Technology (NTNU), Trondheim, Norway

[5]Department of Cancer Research and Molecular Medicine and PROMEC Core Facility for Proteomics and Modomics, Norwegian University of Science and Technology NTNU, and the Central Norway Regional Health Authority Norway.

[6]Department of Physiology & Pharmacology, The University of Toledo, Toledo, OH

[7]Department of Anesthesiology, University of Michigan Medical School, Ann Arbor, Michigan, USA

[8]Department of Molecular and Integrative Physiology, University of Michigan, Ann Arbor, MI

[9]School of Human Movement & Nutrition Sciences, University of Queensland, Australia

***Corresponding author**

Av. Prof. Mello Moraes, 65, São Paulo, Brazil, PO Box 05508-030

Telephone/FAX: +55 11 3091 8732 / +55 11 3813 5921

Email: pcbrum@usp.

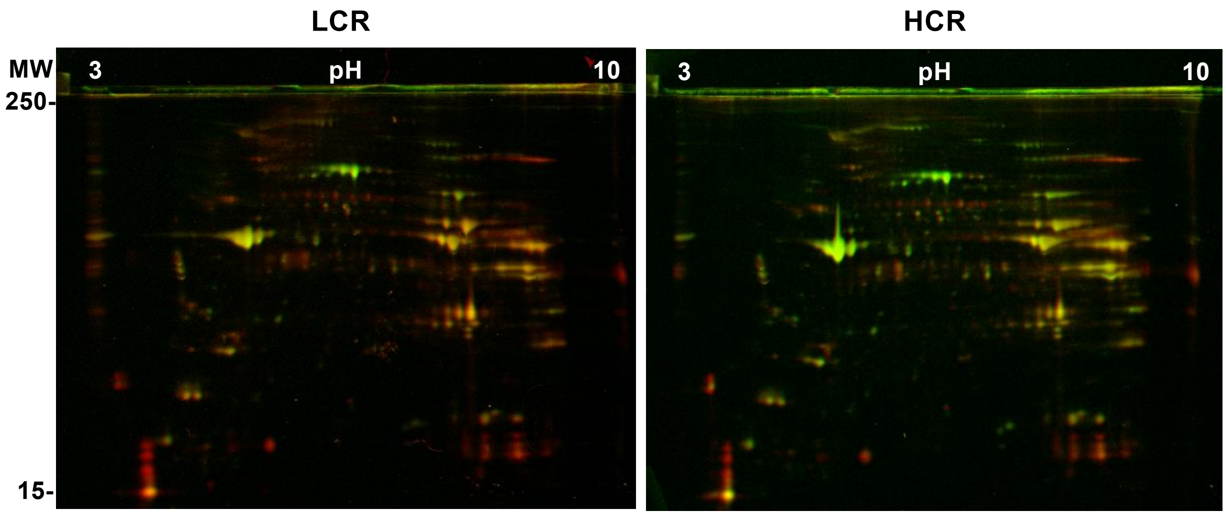

Supplementary Figure 1. Example of the fluorescent two-dimensional gel eletrophoresis (2D-GE) of *plantaris* muscle proteins to visualize and compare cysteine (Cys) redox state between LCR and HCR rats. After 2D- GE separation, the ratio of the intensity between the two fluorophores (oxidized:reduced Cys residues) at each spot reflects the Cys residues redox state of corresponding protein. The infrared scanning of fluorescent gels revealed a more oxidized redox status of HCR *plantaris* (merging signals show the spots with a greener intensity) proteins when compared with LCR.

Plantaris muscle

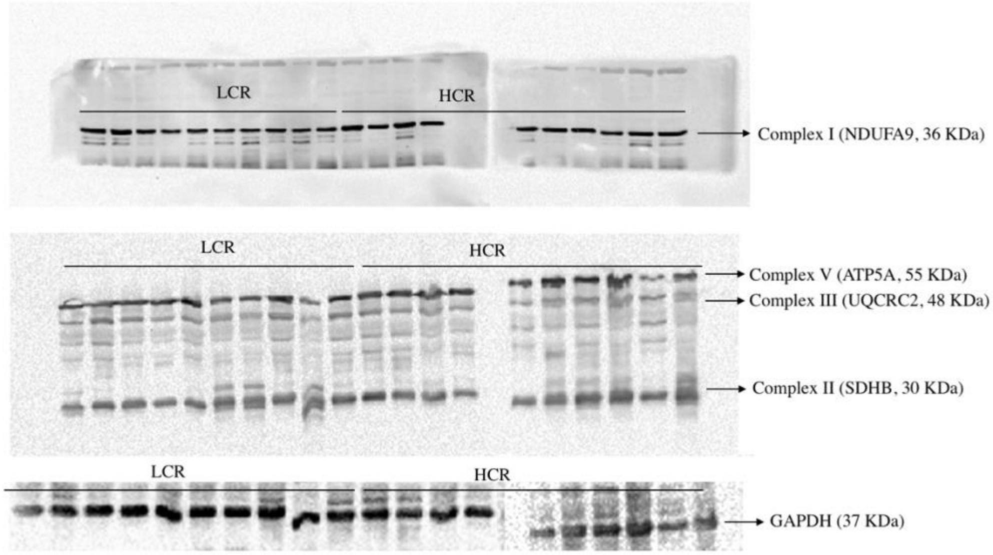

Supplementary Figure 2. ***Plantaris*** **muscle western blot in HCR and LCR animals.** Mitochondrial complex I was measured using Anti-NDUFA9 antibody [20C11B11B11] ab14713 (Abcam, #14713), mitochondrial complexes II, III and V were measured using Total Oxphos Rodent WB antibody cocktail (MitoSciences/Abcam, #MS604/ab110413). GAPDH (Santa Cruz, #sc-20358)

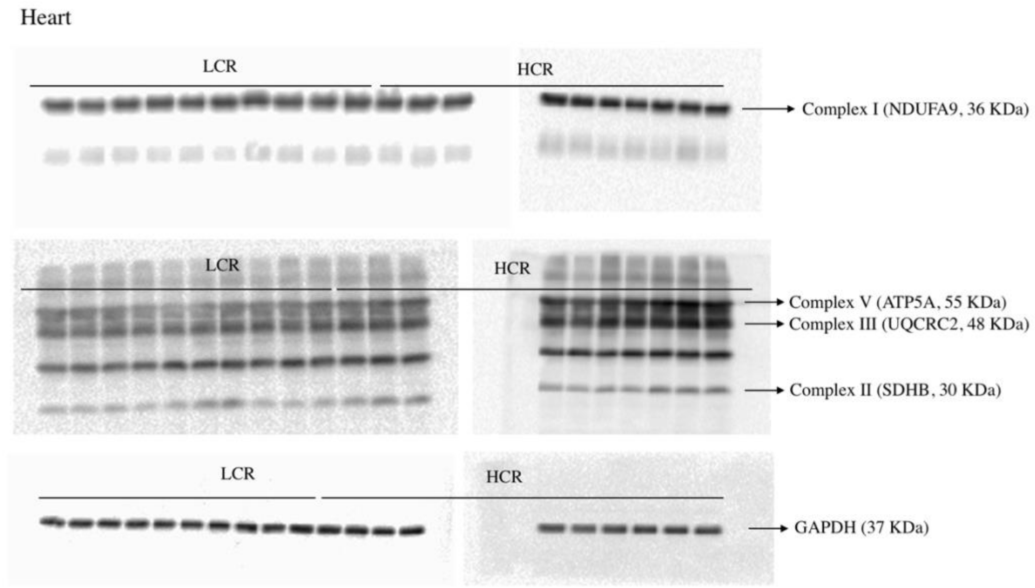

Supplementary Figure 3. Heart western blot in HCR and LCR animals. Mitochondrial complex I was measured using Anti-NDUFA9 antibody [20C11B11B11] ab14713 (Abcam, #14713), mitochondrial complexes II, III and V were measured using Total Oxphos Rodent WB antibody cocktail (MitoSciences/Abcam, #MS604/ab110413). GAPDH (Santa Cruz, #sc-20358).

Table S1. Dobutamine-stress echocardiographic data in LCR and HCR animals.

	Dose 1		Dose 2		Dose 3	
	LCR	HCR	LCR	HCR	LCR	HCR
HR (bpm)	464.33 ± 11.32	461.11 ± 7.28	473.67 ± 16.73	478.22 ± 7.17	466.44 ± 20.40	468.33 ± 15.09
LVAWd (mm)	1.51 ± 0.17	1.46 ± 0.14	1.54 ± 0.17	1.37 ± 0.19	1.42 ± 0.17	1.72 ± 0.09
LVAWs (mm)	2.95 ± 0.15	2.92 ± 0.12	2.96 ± 0.15	3.03 ± 0.14	2.70 ± 0.18	3.10 ± 0.11
LVEDD (mm)	5.30 ± 0.23	5.11 ± 0.26	5.22 ± 0.15	5.28 ± 0.28	5.59 ± 0.25	5.49 ± 0.25
LVESD (mm)	1.64 ± 0.27	1.77 ± 0.20	1.74 ± 0.16	1.42 ± 0.24	2.13 ± 0.31	1.43 ± 0.22
FS (%)	62.11 ± 3.03	63.78 ± 1.77	63.78 ± 1.69	65.10 ± 1.77	60 ± 3.52	68.36 ± 2.25
EF (%)	89.33 ± 2.05	90.87 ± 1.33	91.11 ± 0.96	91.33 ± 1.33	87.63 ± 2.59	93.05 ± 1.48

HR, heart rate; LVAWd and LVAWs, LV anterior wall thickness in diastole and sistole, respectively; LVEDD, LV end-diastolic diameter, and LVESD, LV end-systolic diameter. Data are expressed as mean ± SEM. n = 10 per group.

Table S2. List of Proteins Containing Redox-Sensitive Cys Residues in the *plantaris* muscle of LCR and HCR rats

PLANTARIS*

Accession	Description	HCR	LCR	Fold-Change HCR / LCR	Log2 Fold-change
O88989	Malate dehydrogenase, cytoplasmic OS=Rattus norvegicus GN=Mdh1 PE=1 SV=3 - [MDHC_RAT]	89.22	2.78	32.12	**5.01**
P12007	Isovaleryl-CoA dehydrogenase, mitochondrial OS=Rattus norvegicus GN=Ivd PE=1 SV=2 - [IVD_RAT]	144.58	4.54	31.86	**4.99**
P11507-2	Isoform SERCA2A of Sarcoplasmic/endoplasmic reticulum calcium ATPase 2 OS=Rattus norvegicus GN=Atp2a2 - [AT2A2_RAT]	71.73	2.45	29.27	**4.87**
P48675	Desmin OS=Rattus norvegicus GN=Des PE=1 SV=2 - [DESM_RAT]	73.42	2.69	27.31	**4.77**
G3V936	Citrate synthase OS=Rattus norvegicus GN=Cs PE=3 SV=1 - [G3V936_RAT]	47.86	1.79	26.67	**4.74**
P85834	Elongation factor Tu, mitochondrial OS=Rattus norvegicus GN=Tufm PE=1 SV=1 - [EFTU_RAT]	109.33	4.31	25.35	**4.66**
M0R629	Adenylosuccinate synthetase isozyme 1 OS=Rattus norvegicus GN=Adssl1 PE=3 SV=1 - [M0R629_RAT]	56.76	2.30	24.63	**4.62**
D4AEH9	Amylo-1, 6-glucosidase, 4-alpha-glucanotransferase (Glycogen debranching enzyme, glycogen storage disease type III) (Predicted), isoform CRA_a OS=Rattus norvegicus GN=Agl PE=4 SV=1 - [D4AEH9_RAT]	55.00	2.24	24.59	**4.62**
B4F7C2	Protein Tubb4a OS=Rattus norvegicus GN=Tubb4a PE=2 SV=1 - [B4F7C2_RAT]	42.93	1.83	23.47	**4.55**
Q6P6R2	Dihydrolipoyl dehydrogenase, mitochondrial OS=Rattus norvegicus GN=Dld PE=1 SV=1 - [DLDH_RAT]	116.85	5.10	22.91	**4.52**
P17764	Acetyl-CoA acetyltransferase, mitochondrial OS=Rattus norvegicus GN=Acat1 PE=1 SV=1 - [THIL_RAT]	99.12	4.36	22.75	**4.51**
Q9ER34	Aconitate hydratase, mitochondrial OS=Rattus norvegicus GN=Aco2 PE=1 SV=2 - [ACON_RAT]	63.42	2.80	22.61	**4.50**
D3ZA38	Myosin binding protein C, fast-type (Predicted) OS=Rattus norvegicus GN=Mybpc2 PE=4 SV=1 - [D3ZA38_RAT]	50.01	2.23	22.38	**4.48**
Q68FY0	Cytochrome b-c1 complex subunit 1, mitochondrial OS=Rattus norvegicus GN=Uqcrc1 PE=1 SV=1 - [QCR1_RAT]	47.93	2.16	22.23	**4.47**
P13221	Aspartate aminotransferase, cytoplasmic OS=Rattus norvegicus GN=Got1 PE=1 SV=3 - [AATC_RAT]	97.12	4.44	21.88	**4.45**
P16617	Phosphoglycerate kinase 1 OS=Rattus norvegicus GN=Pgk1 PE=1 SV=2 - [PGK1_RAT]	78.86	3.67	21.50	**4.43**
P04642	L-lactate dehydrogenase A chain OS=Rattus norvegicus GN=Ldha PE=1 SV=1 - [LDHA_RAT]	38.33	1.81	21.21	**4.41**
P07943	Aldose reductase OS=Rattus norvegicus GN=Akr1b1 PE=1 SV=3 - [ALDR_RAT]	130.08	6.16	21.12	**4.40**
G3V8V6	O-acetyl-ADP-ribose deacetylase MACROD1 OS=Rattus norvegicus GN=Macrod1 PE=4 SV=2 - [G3V8V6_RAT]	82.72	3.99	20.74	**4.37**
G3V7K1	Myomesin 2 OS=Rattus norvegicus GN=Myom2 PE=4 SV=1 - [G3V7K1_RAT]	50.22	2.44	20.59	**4.36**
P58775	Tropomyosin beta chain OS=Rattus norvegicus GN=Tpm2 PE=2 SV=1 - [TPM2_RAT]	223.76	10.98	20.37	**4.35**
Q6P6V0	Glucose-6-phosphate isomerase OS=Rattus norvegicus GN=Gpi PE=1 SV=1 - [G6PI_RAT]	30.85	1.55	19.88	**4.31**
D3ZCV0	Protein Actn2 OS=Rattus norvegicus GN=Actn2 PE=4 SV=1 - [D3ZCV0_RAT]	32.51	1.64	19.85	**4.31**
P41565	Isocitrate dehydrogenase [NAD] subunit gamma 1, mitochondrial OS=Rattus norvegicus GN=Idh3g PE=2 SV=2 - [IDHG1_RAT]	32.20	1.66	19.37	**4.28**
F1LMU0	Myosin-4 OS=Rattus norvegicus GN=Myh4 PE=2 SV=1 - [F1LMU0_RAT]	77.68	4.17	18.62	**4.22**
D3ZGY4	Glyceraldehyde-3-phosphate dehydrogenase OS=Rattus norvegicus GN=RGD1560797 PE=3 SV=1 - [D3ZGY4_RAT]	36.97	1.99	18.59	**4.22**
P00507	Aspartate aminotransferase, mitochondrial OS=Rattus norvegicus GN=Got2 PE=1 SV=2 - [AATM_RAT]	38.01	2.08	18.25	**4.19**

Q63610-2	Isoform 2 of Tropomyosin alpha-3 chain OS=Rattus norvegicus GN=Tpm3 - [TPM3_RAT]	338.61	18.78	18.03	**4.17**
P18163	Long-chain-fatty-acid--CoA ligase 1 OS=Rattus norvegicus GN=Acsl1 PE=1 SV=1 - [ACSL1_RAT]	42.07	2.38	17.65	**4.14**
Q8R4I6	Actinin alpha 3, isoform CRA_a OS=Rattus norvegicus GN=Actn3 PE=2 SV=1 - [Q8R4I6_RAT]	30.32	1.73	17.54	**4.13**
A0JPQ4	Tripartite motif-containing protein 72 OS=Rattus norvegicus GN=Trim72 PE=2 SV=1 - [TRI72_RAT]	83.53	4.78	17.47	**4.13**
D4A2S4	Myosin-binding protein C, slow-type OS=Rattus norvegicus GN=Mybpc1 PE=4 SV=2 - [D4A2S4_RAT]	44.20	2.54	17.40	**4.12**
P62634	Cellular nucleic acid-binding protein OS=Rattus norvegicus GN=Cnbp PE=2 SV=1 - [CNBP_RAT]	234.29	13.56	17.27	**4.11**
D4A4B0	Uncharacterized protein (Fragment) OS=Rattus norvegicus PE=4 SV=2 - [D4A4B0_RAT]	31.64	1.89	16.72	**4.06**
D3ZQ55	Junctophilin 1 (Predicted) OS=Rattus norvegicus GN=Jph1 PE=4 SV=1 - [D3ZQ55_RAT]	48.08	2.88	16.72	**4.06**
Q66HS7	PDZ and LIM domain protein 3 OS=Rattus norvegicus GN=Pdlim3 PE=1 SV=2 - [PDLI3_RAT]	163.21	9.87	16.54	**4.05**
P04636	Malate dehydrogenase, mitochondrial OS=Rattus norvegicus GN=Mdh2 PE=1 SV=2 - [MDHM_RAT]	110.27	6.77	16.28	**4.03**
Q64578	Sarcoplasmic/endoplasmic reticulum calcium ATPase 1 OS=Rattus norvegicus GN=Atp2a1 PE=2 SV=1 - [AT2A1_RAT]	30.31	1.89	16.00	**4.00**
P08461	Dihydrolipoyllysine-residue acetyltransferase component of pyruvate dehydrogenase complex, mitochondrial OS=Rattus norvegicus GN=Dlat PE=1 SV=3 - [ODP2_RAT]	51.99	3.32	15.66	**3.97**
P08009	Glutathione S-transferase Yb-3 OS=Rattus norvegicus GN=Gstm3 PE=1 SV=2 - [GSTM4_RAT]	89.96	5.75	15.66	**3.97**
G3V7U0	Cysteine and glycine-rich protein 3 OS=Rattus norvegicus GN=Csrp3 PE=4 SV=1 - [G3V7U0_RAT]	87.52	5.69	15.39	**3.94**
R4GNK3	Thioredoxin (Fragment) OS=Rattus norvegicus GN=Txn1 PE=3 SV=1 - [R4GNK3_RAT]	89.30	5.93	15.07	**3.91**
P26284	Pyruvate dehydrogenase E1 component subunit alpha, somatic form, mitochondrial OS=Rattus norvegicus GN=Pdha1 PE=1 SV=2 - [ODPA_RAT]	38.36	2.62	14.64	**3.87**
Q9WVK7	Hydroxyacyl-coenzyme A dehydrogenase, mitochondrial OS=Rattus norvegicus GN=Hadh PE=2 SV=1 - [HCDH_RAT]	37.36	2.60	14.34	**3.84**
P05065	Fructose-bisphosphate aldolase A OS=Rattus norvegicus GN=Aldoa PE=1 SV=2 - [ALDOA_RAT]	63.59	4.50	14.13	**3.82**
P07895	Superoxide dismutase [Mn], mitochondrial OS=Rattus norvegicus GN=Sod2 PE=1 SV=2 - [SODM_RAT]	141.28	10.00	14.13	**3.82**
P15429	Beta-enolase OS=Rattus norvegicus GN=Eno3 PE=1 SV=3 - [ENOB_RAT]	74.39	5.28	14.08	**3.82**
Q499Q4	Phosphoglucomutase 1 OS=Rattus norvegicus GN=Pgm1 PE=2 SV=2 - [Q499Q4_RAT]	24.18	1.72	14.05	**3.81**
P00564	Creatine kinase M-type OS=Rattus norvegicus GN=Ckm PE=1 SV=2 - [KCRM_RAT]	75.81	5.42	13.99	**3.81**
P14604	Enoyl-CoA hydratase, mitochondrial OS=Rattus norvegicus GN=Echs1 PE=1 SV=1 - [ECHM_RAT]	53.76	4.05	13.27	**3.73**
D3ZF13	Acyl carrier protein OS=Rattus norvegicus GN=LOC683884 PE=3 SV=1 - [D3ZF13_RAT]	110.23	8.40	13.12	**3.71**
Q6PDW8	Glutathione peroxidase OS=Rattus norvegicus GN=Gpx1 PE=2 SV=1 - [Q6PDW8_RAT]	48.48	3.84	12.62	**3.66**
G3V8V3	Phosphorylase OS=Rattus norvegicus GN=Pygm PE=3 SV=1 - [G3V8V3_RAT]	25.00	2.04	12.25	**3.61**
P19804	Nucleoside diphosphate kinase B OS=Rattus norvegicus GN=Nme2 PE=1 SV=1 - [NDKB_RAT]	89.55	7.34	12.21	**3.61**
D3ZPF0	Four and a half LIM domains 3 (Predicted) OS=Rattus norvegicus GN=Fhl3 PE=4 SV=1 - [D3ZPF0_RAT]	100.28	8.60	11.66	**3.54**
P48500	Triosephosphate isomerase OS=Rattus norvegicus GN=Tpi1 PE=1 SV=2 - [TPIS_RAT]	79.20	7.15	11.07	**3.47**
Q304F3	Protein Tnnc2 OS=Rattus norvegicus GN=Tnnc2 PE=2 SV=1 - [Q304F3_RAT]	491.77	44.72	11.00	**3.46**
G3V8B0	Myosin-7 OS=Rattus norvegicus GN=Myh7 PE=4 SV=1 - [G3V8B0_RAT]	56.98	5.21	10.94	**3.45**
Q9Z2L0	Voltage-dependent anion-selective channel protein 1 OS=Rattus norvegicus GN=Vdac1 PE=1 SV=4 - [VDAC1_RAT]	44.92	4.15	10.83	**3.44**
Q6P792	Four and a half LIM domains 1 OS=Rattus norvegicus GN=Fhl1 PE=2 SV=1 - [Q6P792_RAT]	63.56	6.06	10.49	**3.39**
D3ZWT8	Uncharacterized protein OS=Rattus norvegicus GN=Atp5h PE=4 SV=2 - [D3ZWT8_RAT]	152.37	14.67	10.39	**3.38**
D3ZHA0	Protein Flnc OS=Rattus norvegicus GN=Flnc PE=4 SV=1 - [D3ZHA0_RAT]	45.88	4.44	10.34	**3.37**

P15650	Long-chain specific acyl-CoA dehydrogenase, mitochondrial OS=Rattus norvegicus GN=Acadl PE=1 SV=1 - [ACADL_RAT]	24.72	2.49	9.94	**3.31**
P11980	Pyruvate kinase PKM OS=Rattus norvegicus GN=Pkm PE=1 SV=3 - [KPYM_RAT]	21.57	2.25	9.60	**3.26**
P16290	Phosphoglycerate mutase 2 OS=Rattus norvegicus GN=Pgam2 PE=2 SV=2 - [PGAM2_RAT]	30.53	3.21	9.51	**3.25**
F8WG17	Troponin I, fast skeletal muscle (Fragment) OS=Rattus norvegicus GN=Tnni2 PE=4 SV=2 - [F8WG17_RAT]	59.28	6.34	9.34	**3.22**
D3ZCZ9	Protein LOC100912599 OS=Rattus norvegicus GN=LOC100912599 PE=4 SV=1 - [D3ZCZ9_RAT]	77.75	8.32	9.34	**3.22**
P11762	Galectin-1 OS=Rattus norvegicus GN=Lgals1 PE=1 SV=2 - [LEG1_RAT]	119.86	14.09	8.51	**3.09**
Q6AYF2	LIM and cysteine-rich domains 1 OS=Rattus norvegicus GN=Lmcd1 PE=2 SV=1 - [Q6AYF2_RAT]	61.05	7.19	8.49	**3.09**
Q7TP78	Aa2-258 OS=Rattus norvegicus GN=Ndufa8 PE=2 SV=1 - [Q7TP78_RAT]	126.68	15.12	8.38	**3.07**
B0K010	Protein Txndc17 OS=Rattus norvegicus GN=Txndc17 PE=2 SV=1 - [B0K010_RAT]	25.95	3.16	8.22	**3.04**
P24090	Alpha-2-HS-glycoprotein OS=Rattus norvegicus GN=Ahsg PE=1 SV=2 - [FETUA_RAT]	535.65	66.56	8.05	**3.01**
Q05962	ADP/ATP translocase 1 OS=Rattus norvegicus GN=Slc25a4 PE=1 SV=3 - [ADT1_RAT]	8.24	1.04	7.95	**2.99**
Q5U2U8	Bcl2-associated athanogene 3 OS=Rattus norvegicus GN=Bag3 PE=2 SV=1 - [Q5U2U8_RAT]	169.24	21.37	7.92	**2.99**
D3Z874	Uncharacterized protein OS=Rattus norvegicus PE=4 SV=1 - [D3Z874_RAT]	27.12	3.45	7.86	**2.97**
Q9QZ76	Myoglobin OS=Rattus norvegicus GN=Mb PE=1 SV=3 - [MYG_RAT]	75.39	9.77	7.72	**2.95**
O88767	Protein DJ-1 OS=Rattus norvegicus GN=Park7 PE=1 SV=1 - [PARK7_RAT]	51.56	7.06	7.31	**2.87**
P20059	Hemopexin OS=Rattus norvegicus GN=Hpx PE=1 SV=3 - [HEMO_RAT]	189.82	28.96	6.55	**2.71**
B4F789	Apolipoprotein B editing complex 2 (Predicted), isoform CRA_a OS=Rattus norvegicus GN=Apobec2 PE=2 SV=1 - [B4F789_RAT]	30.26	4.66	6.49	**2.70**
P12346	Serotransferrin OS=Rattus norvegicus GN=Tf PE=1 SV=3 - [TRFE_RAT]	159.50	24.93	6.40	**2.68**
F1M8C9	Uncharacterized protein (Fragment) OS=Rattus norvegicus PE=4 SV=2 - [F1M8C9_RAT]	16.02	2.53	6.34	**2.67**
P68136	Actin, alpha skeletal muscle OS=Rattus norvegicus GN=Acta1 PE=1 SV=1 - [ACTS_RAT]	23.99	3.80	6.31	**2.66**
Q66HF1	NADH-ubiquinone oxidoreductase 75 kDa subunit, mitochondrial OS=Rattus norvegicus GN=Ndufs1 PE=1 SV=1 - [NDUS1_RAT]	38.16	6.05	6.30	**2.66**
P02091	Hemoglobin subunit beta-1 OS=Rattus norvegicus GN=Hbb PE=1 SV=3 - [HBB1_RAT]	53.45	9.33	5.73	**2.52**
P04466	Myosin regulatory light chain 2, skeletal muscle isoform OS=Rattus norvegicus GN=Mylpf PE=2 SV=2 - [MLRS_RAT]	38.98	7.46	5.23	**2.39**
F1M953	Stress-70 protein, mitochondrial OS=Rattus norvegicus GN=Hspa9 PE=3 SV=1 - [F1M953_RAT]	28.02	5.42	5.17	**2.37**
F1M978	Inositol monophosphatase 1 OS=Rattus norvegicus GN=Impa1 PE=4 SV=1 - [F1M978_RAT]	35.41	6.97	5.08	**2.34**
P60711	Actin, cytoplasmic 1 OS=Rattus norvegicus GN=Actb PE=1 SV=1 - [ACTB_RAT]	19.71	3.92	5.03	**2.33**
Q5M9I5	Cytochrome b-c1 complex subunit 6, mitochondrial OS=Rattus norvegicus GN=Uqcrh PE=3 SV=1 - [QCR6_RAT]	120.14	24.48	4.91	**2.29**
P13086	Succinyl-CoA ligase [ADP/GDP-forming] subunit alpha, mitochondrial OS=Rattus norvegicus GN=Suclg1 PE=2 SV=2 - [SUCA_RAT]	18.48	3.93	4.70	**2.23**
P10111	Peptidyl-prolyl cis-trans isomerase A OS=Rattus norvegicus GN=Ppia PE=1 SV=2 - [PPIA_RAT]	38.61	8.46	4.56	**2.19**
Q4PP99	Cardiac troponin C OS=Rattus norvegicus GN=Tnnc1 PE=2 SV=1 - [Q4PP99_RAT]	65.34	15.33	4.26	**2.09**
Q6P7Q4	Lactoylglutathione lyase OS=Rattus norvegicus GN=Glo1 PE=1 SV=3 - [LGUL_RAT]	19.21	4.51	4.26	**2.09**
P29117	Peptidyl-prolyl cis-trans isomerase F, mitochondrial OS=Rattus norvegicus GN=Ppif PE=1 SV=2 - [PPIF_RAT]	22.26	5.33	4.18	**2.06**
Q5RK08	Glioblastoma amplified sequence OS=Rattus norvegicus GN=Gbas PE=2 SV=1 - [Q5RK08_RAT]	8.32	2.00	4.16	**2.06**
Q7TP52	Carboxymethylenebutenolidase homolog OS=Rattus norvegicus GN=Cmbl PE=2 SV=1 - [CMBL_RAT]	21.04	5.26	4.00	**2.00**
D4A0T0	Protein Ndufb10 OS=Rattus norvegicus GN=Ndufb10 PE=4 SV=1 - [D4A0T0_RAT]	300.77	91.40	3.29	**1.72**
P16409	Myosin light chain 3 OS=Rattus norvegicus GN=Myl3 PE=2 SV=2 - [MYL3_RAT]	30.16	9.21	3.27	**1.71**

G3V907	Protein LOC100911356 OS=Rattus norvegicus GN=LOC100911356 PE=4 SV=1 - [G3V907_RAT]	14.61	4.47	3.27	**1.71**
F1LNF7	Isocitrate dehydrogenase [NAD] subunit alpha, mitochondrial OS=Rattus norvegicus GN=Idh3a PE=3 SV=1 - [F1LNF7_RAT]	17.48	5.41	3.23	**1.69**
P13803	Electron transfer flavoprotein subunit alpha, mitochondrial OS=Rattus norvegicus GN=Etfa PE=1 SV=4 - [ETFA_RAT]	14.54	4.64	3.13	**1.65**
D3ZWV7	Succinate-semialdehyde dehydrogenase, mitochondrial OS=Rattus norvegicus GN=Aldh5a1 PE=2 SV=1 - [D3ZWV7_RAT]	14.76	4.79	3.08	**1.62**
Q561S0	NADH dehydrogenase [ubiquinone] 1 alpha subcomplex subunit 10, mitochondrial OS=Rattus norvegicus GN=Ndufa10 PE=1 SV=1 - [NDUAA_RAT]	7.61	2.48	3.07	**1.62**
P80254	D-dopachrome decarboxylase OS=Rattus norvegicus GN=Ddt PE=1 SV=3 - [DOPD_RAT]	20.67	7.28	2.84	**1.50**
Q920L2	Succinate dehydrogenase [ubiquinone] flavoprotein subunit, mitochondrial OS=Rattus norvegicus GN=Sdha PE=1 SV=1 - [DHSA_RAT]	8.76	3.41	2.57	**1.36**
P07154	Cathepsin L1 OS=Rattus norvegicus GN=Ctsl PE=1 SV=2 - [CATL1_RAT]	31.95	16.17	1.98	**0.98**
P09605	Creatine kinase S-type, mitochondrial OS=Rattus norvegicus GN=Ckmt2 PE=1 SV=2 - [KCRS_RAT]	5.00	2.68	1.86	**0.90**
P02770	Serum albumin OS=Rattus norvegicus GN=Alb PE=1 SV=2 - [ALBU_RAT]	551.11	303.05	1.82	**0.86**
D4ABA9	Protein LOC100910104 OS=Rattus norvegicus GN=LOC100910104 PE=4 SV=1 - [D4ABA9_RAT]	12.78	7.37	1.74	**0.80**
P02600	Myosin light chain 1/3, skeletal muscle isoform OS=Rattus norvegicus GN=Myl1 PE=1 SV=2 - [MYL1_RAT]	7.92	5.89	1.34	**0.43**
B0BNE5	S-formylglutathione hydrolase OS=Rattus norvegicus GN=Esd PE=1 SV=1 - [ESTD_RAT]	2.95	2.58	1.14	**0.19**
P63255	Cysteine-rich protein 1 OS=Rattus norvegicus GN=Crip1 PE=1 SV=2 - [CRIP1_RAT]	2.80	2.60	1.07	**0.10**
D4AAB5	Protein Pm20d2 OS=Rattus norvegicus GN=Pm20d2 PE=4 SV=1 - [D4AAB5_RAT]	4.47	5.00	0.89	**-0.16**
D3ZM60	Protein Ppp1r3a OS=Rattus norvegicus GN=Ppp1r3a PE=4 SV=1 - [D3ZM60_RAT]	3.88	4.37	0.89	**-0.17**
A0JN30	Canopy 2 homolog (Zebrafish) OS=Rattus norvegicus GN=Cnpy2 PE=2 SV=1 - [A0JN30_RAT]	4.41	5.83	0.76	**-0.40**
D3ZDQ9	Protein Sgca OS=Rattus norvegicus GN=Sgca PE=4 SV=1 - [D3ZDQ9_RAT]	4.33	7.01	0.62	**-0.70**
P46462	Transitional endoplasmic reticulum ATPase OS=Rattus norvegicus GN=Vcp PE=1 SV=3 - [TERA_RAT]	0.40	0.66	0.60	**-0.73**
F1M614	Protein Lama2 (Fragment) OS=Rattus norvegicus GN=Lama2 PE=4 SV=2 - [F1M614_RAT]	486.71	856.03	0.57	**-0.81**
Q52KJ8	Methionine-R-sulfoxide reductase B1 OS=Rattus norvegicus GN=Msrb1 PE=3 SV=2 - [MSRB1_RAT]	7.73	16.33	0.47	**-1.08**
Q641Y2	NADH dehydrogenase [ubiquinone] iron-sulfur protein 2, mitochondrial OS=Rattus norvegicus GN=Ndufs2 PE=1 SV=1 - [NDUS2_RAT]	0.88	1.86	0.47	**-1.08**
P04692	Tropomyosin alpha-1 chain OS=Rattus norvegicus GN=Tpm1 PE=1 SV=3 - [TPM1_RAT]	7.29	16.21	0.45	**-1.15**
B0BN52	Mitochondrial carrier homolog 2 (C. elegans) OS=Rattus norvegicus GN=Mtch2 PE=2 SV=1 - [B0BN52_RAT]	0.21	1.88	0.11	**-3.19**
P02767	Transthyretin OS=Rattus norvegicus GN=Ttr PE=1 SV=1 - [TTHY_RAT]	2.52	33.55	0.07	**-3.74**

* Table includes the redox ratio of individual Cys residues within those proteins. The redox state of selected redox Cys residues labeled with both light ^{12}C-form and heavy ^{13}C-form was calculated using the oxidation ratio (oxidized:reduced).

Table S3. List of Proteins Containing Redox-Sensitive Cys Residues in the heart of LCR and HCR rats.

		HEART*			
**Accession**	**Description**	**HCR**	**LCR**	**Fold-Change HCR / LCR**	**Log2 Fold-change**
Q5XIN6	LETM1 and EF-hand domain-containing protein 1, mitochondrial OS=Rattus norvegicus GN=Letm1 PE=1 SV=1 - [LETM1_RAT]	10.62	3.36	3.16	**1.66**
Q6AXV4	Sorting and assembly machinery component 50 homolog OS=Rattus norvegicus GN=Samm50 PE=1 SV=1 - [SAM50_RAT]	1.00	0.32	3.11	**1.64**
P00564	Creatine kinase M-type OS=Rattus norvegicus GN=Ckm PE=1 SV=2 - [KCRM_RAT]	1.37	0.54	2.53	**1.34**
P20059	Hemopexin OS=Rattus norvegicus GN=Hpx PE=1 SV=3 - [HEMO_RAT]	6.01	3.85	1.56	**0.64**
Q5BJQ0	Chaperone activity of bc1 complex-like, mitochondrial OS=Rattus norvegicus GN=Adck3 PE=2 SV=1 - [ADCK3_RAT]	0.55	0.36	1.54	**0.62**
Q5BJZ3	Nicotinamide nucleotide transhydrogenase OS=Rattus norvegicus GN=Nnt PE=2 SV=1 - [Q5BJZ3_RAT]	0.75	0.51	1.47	**0.56**
P62074	Mitochondrial import inner membrane translocase subunit Tim10 OS=Rattus norvegicus GN=Timm10 PE=3 SV=1 - [TIM10_RAT]	142.46	98.86	1.44	**0.53**
Q5XI72	Eukaryotic translation initiation factor 4H OS=Rattus norvegicus GN=Eif4h PE=1 SV=1 - [IF4H_RAT]	0.88	0.63	1.40	**0.49**
Q5M9I5	Cytochrome b-c1 complex subunit 6, mitochondrial OS=Rattus norvegicus GN=Uqcrh PE=3 SV=1 - [QCR6_RAT]	3.79	2.75	1.38	**0.46**
D3ZHA0	Protein Flnc OS=Rattus norvegicus GN=Flnc PE=2 SV=1 - [D3ZHA0_RAT]	0.83	0.62	1.33	**0.41**
Q6P6R2	Dihydrolipoyl dehydrogenase, mitochondrial OS=Rattus norvegicus GN=Dld PE=1 SV=1 - [DLDH_RAT]	0.78	0.63	1.23	**0.30**
B0LPN4-2	Isoform 2 of Ryanodine receptor 2 OS=Rattus norvegicus GN=Ryr2 - [RYR2_RAT]	0.65	0.54	1.21	**0.27**
E9PST1	Protein RGD1310507 OS=Rattus norvegicus GN=RGD1310507 PE=2 SV=2 - [E9PST1_RAT]	1.24	1.03	1.20	**0.26**
P34058	Heat shock protein HSP 90-beta OS=Rattus norvegicus GN=Hsp90ab1 PE=1 SV=4 - [HS90B_RAT]	0.47	0.40	1.19	**0.25**
P06685	Sodium/potassium-transporting ATPase subunit alpha-1 OS=Rattus norvegicus GN=Atp1a1 PE=1 SV=1 - [AT1A1_RAT]	0.45	0.39	1.15	**0.21**
Q9EPH1-2	Isoform 2 of Alpha-1B-glycoprotein OS=Rattus norvegicus GN=A1bg - [A1BG_RAT]	1.59	1.41	1.13	**0.17**
D4A4B0	Uncharacterized protein (Fragment) OS=Rattus norvegicus PE=2 SV=2 - [D4A4B0_RAT]	0.56	0.52	1.09	**0.12**
O35115	Four and a half LIM domains protein 2 OS=Rattus norvegicus GN=Fhl2 PE=1 SV=1 - [FHL2_RAT]	1.33	1.24	1.08	**0.11**
P68511	14-3-3 protein eta OS=Rattus norvegicus GN=Ywhah PE=1 SV=2 - [1433F_RAT]	0.41	0.38	1.06	**0.09**
P14562	Lysosome-associated membrane glycoprotein 1 OS=Rattus norvegicus GN=Lamp1 PE=1 SV=1 - [LAMP1_RAT]	18.17	17.61	1.03	**0.05**
F1LMV6	Protein Dsp OS=Rattus norvegicus GN=Dsp PE=2 SV=1 - [F1LMV6_RAT]	1.44	1.40	1.02	**0.03**
Q920L2	Succinate dehydrogenase [ubiquinone] flavoprotein subunit, mitochondrial OS=Rattus norvegicus GN=Sdha PE=1 SV=1 - [DHSA_RAT]	0.61	0.60	1.02	**0.03**
P41562	Isocitrate dehydrogenase [NADP] cytoplasmic OS=Rattus norvegicus GN=Idh1 PE=1 SV=1 - [IDHC_RAT]	0.68	0.69	0.98	**-0.03**
Q9QZ76	Myoglobin OS=Rattus norvegicus GN=Mb PE=1 SV=3 - [MYG_RAT]	0.67	0.70	0.97	**-0.05**
Q7TMC7	Ab2-417 OS=Rattus norvegicus GN=Tf PE=2 SV=1 - [Q7TMC7_RAT]	5.00	5.27	0.95	**-0.08**
P14046	Alpha-1-inhibitor 3 OS=Rattus norvegicus GN=A1i3 PE=1 SV=1 - [A1I3_RAT]	8.93	9.42	0.95	**-0.08**
P97576	GrpE protein homolog 1, mitochondrial OS=Rattus norvegicus GN=Grpel1 PE=1 SV=2 - [GRPE1_RAT]	1.52	1.61	0.94	**-0.08**
D3ZUL3	Protein Col6a1 OS=Rattus norvegicus GN=Col6a1 PE=4 SV=1 - [D3ZUL3_RAT]	2.53	2.70	0.94	**-0.10**
B0BNE5	S-formylglutathione hydrolase OS=Rattus norvegicus GN=Esd PE=1 SV=1 - [ESTD_RAT]	1.05	1.13	0.93	**-0.11**

P49134	Integrin beta-1 OS=Rattus norvegicus GN=Itgb1 PE=2 SV=1 - [ITB1_RAT]	9.61	10.54	0.91	**-0.13**
F1LPC7	Hepatoma-derived growth factor OS=Rattus norvegicus GN=Hdgf PE=2 SV=1 - [F1LPC7_RAT]	1.51	1.69	0.89	**-0.16**
P15650	Long-chain specific acyl-CoA dehydrogenase, mitochondrial OS=Rattus norvegicus GN=Acadl PE=1 SV=1 - [ACADL_RAT]	0.29	0.33	0.89	**-0.17**
P02091	Hemoglobin subunit beta-1 OS=Rattus norvegicus GN=Hbb PE=1 SV=3 - [HBB1_RAT]	0.55	0.62	0.89	**-0.17**
G3V7U4	Lamin-B1 OS=Rattus norvegicus GN=Lmnb1 PE=3 SV=1 - [G3V7U4_RAT]	1.06	1.22	0.87	**-0.20**
Q80W89	NADH dehydrogenase [ubiquinone] 1 alpha subcomplex subunit 11 OS=Rattus norvegicus GN=Ndufa11 PE=2 SV=1 - [NDUAB_RAT]	0.97	1.14	0.85	**-0.24**
Q5XIC0	Enoyl-CoA delta isomerase 2, mitochondrial OS=Rattus norvegicus GN=Eci2 PE=1 SV=1 - [ECI2_RAT]	0.49	0.59	0.84	**-0.25**
P19804	Nucleoside diphosphate kinase B OS=Rattus norvegicus GN=Nme2 PE=1 SV=1 - [NDKB_RAT]	0.40	0.47	0.84	**-0.25**
P42123	L-lactate dehydrogenase B chain OS=Rattus norvegicus GN=Ldhb PE=1 SV=2 - [LDHB_RAT]	0.39	0.47	0.83	**-0.27**
B0BN81	Ribosomal protein S5, isoform CRA_b OS=Rattus norvegicus GN=Rps5 PE=2 SV=1 - [B0BN81_RAT]	0.49	0.59	0.83	**-0.27**
P13803	Electron transfer flavoprotein subunit alpha, mitochondrial OS=Rattus norvegicus GN=Etfa PE=1 SV=4 - [ETFA_RAT]	1.03	1.25	0.82	**-0.28**
P35738	2-oxoisovalerate dehydrogenase subunit beta, mitochondrial OS=Rattus norvegicus GN=Bckdhb PE=1 SV=3 - [ODBB_RAT]	0.38	0.46	0.81	**-0.30**
P32551	Cytochrome b-c1 complex subunit 2, mitochondrial OS=Rattus norvegicus GN=Uqcrc2 PE=1 SV=2 - [QCR2_RAT]	0.45	0.56	0.81	**-0.30**
O88767	Protein DJ-1 OS=Rattus norvegicus GN=Park7 PE=1 SV=1 - [PARK7_RAT]	1.72	2.13	0.81	**-0.30**
P56574	Isocitrate dehydrogenase [NADP], mitochondrial OS=Rattus norvegicus GN=Idh2 PE=1 SV=2 - [IDHP_RAT]	0.28	0.35	0.80	**-0.33**
P49432	Pyruvate dehydrogenase E1 component subunit beta, mitochondrial OS=Rattus norvegicus GN=Pdhb PE=1 SV=2 - [ODPB_RAT]	0.44	0.56	0.79	**-0.34**
P00507	Aspartate aminotransferase, mitochondrial OS=Rattus norvegicus GN=Got2 PE=1 SV=2 - [AATM_RAT]	0.51	0.65	0.79	**-0.34**
H9KVF5	NADH-ubiquinone oxidoreductase chain 3 (Fragment) OS=Rattus norvegicus GN=7066254 PE=3 SV=1 - [H9KVF5_RAT]	0.45	0.58	0.79	**-0.35**
Q5XIH3	NADH dehydrogenase (Ubiquinone) flavoprotein 1 OS=Rattus norvegicus GN=Ndufv1 PE=2 SV=1 - [Q5XIH3_RAT]	0.60	0.77	0.78	**-0.35**
P07340	Sodium/potassium-transporting ATPase subunit beta-1 OS=Rattus norvegicus GN=Atp1b1 PE=1 SV=1 - [AT1B1_RAT]	5.03	6.53	0.77	**-0.38**
P61983	14-3-3 protein gamma OS=Rattus norvegicus GN=Ywhag PE=1 SV=2 - [1433G_RAT]	0.59	0.78	0.76	**-0.40**
P60711	Actin, cytoplasmic 1 OS=Rattus norvegicus GN=Actb PE=1 SV=1 - [ACTB_RAT]	0.57	0.77	0.75	**-0.41**
Q4V8F9	Hydroxysteroid dehydrogenase-like protein 2 OS=Rattus norvegicus GN=Hsdl2 PE=2 SV=1 - [HSDL2_RAT]	0.72	0.97	0.74	**-0.43**
P04636	Malate dehydrogenase, mitochondrial OS=Rattus norvegicus GN=Mdh2 PE=1 SV=2 - [MDHM_RAT]	0.79	1.07	0.74	**-0.43**
P13221	Aspartate aminotransferase, cytoplasmic OS=Rattus norvegicus GN=Got1 PE=1 SV=3 - [AATC_RAT]	0.43	0.58	0.74	**-0.44**
Q6P0K8	Junction plakoglobin OS=Rattus norvegicus GN=Jup PE=1 SV=1 - [PLAK_RAT]	0.54	0.73	0.73	**-0.45**
F1LM84	Nidogen-1 OS=Rattus norvegicus GN=Nid1 PE=2 SV=1 - [F1LM84_RAT]	9.75	13.33	0.73	**-0.45**
O35244	Peroxiredoxin-6 OS=Rattus norvegicus GN=Prdx6 PE=1 SV=3 - [PRDX6_RAT]	0.66	0.91	0.73	**-0.46**
Q07936	Annexin A2 OS=Rattus norvegicus GN=Anxa2 PE=1 SV=2 - [ANXA2_RAT]	0.31	0.43	0.73	**-0.46**
D3ZWT8	Uncharacterized protein OS=Rattus norvegicus GN=Atp5h PE=4 SV=2 - [D3ZWT8_RAT]	2.42	3.36	0.72	**-0.47**
Q9WVK7	Hydroxyacyl-coenzyme A dehydrogenase, mitochondrial OS=Rattus norvegicus GN=Hadh PE=2 SV=1 - [HCDH_RAT]	0.46	0.65	0.72	**-0.48**
Q561S0	NADH dehydrogenase [ubiquinone] 1 alpha subcomplex subunit 10, mitochondrial OS=Rattus norvegicus GN=Ndufa10 PE=1 SV=1 - [NDUAA_RAT]	0.94	1.33	0.71	**-0.50**

F1M614	Protein Lama2 (Fragment) OS=Rattus norvegicus GN=Lama2 PE=2 SV=2 - [F1M614_RAT]	5.09	7.20	0.71	**-0.50**
P15651	Short-chain specific acyl-CoA dehydrogenase, mitochondrial OS=Rattus norvegicus GN=Acads PE=1 SV=2 - [ACADS_RAT]	0.46	0.66	0.70	**-0.51**
G3V741	Phosphate carrier protein, mitochondrial OS=Rattus norvegicus GN=Slc25a3 PE=3 SV=1 - [G3V741_RAT]	0.27	0.39	0.70	**-0.52**
Q7TP78	Aa2-258 OS=Rattus norvegicus GN=Ndufa8 PE=2 SV=1 - [Q7TP78_RAT]	2.49	3.58	0.69	**-0.53**
O70593	Small glutamine-rich tetratricopeptide repeat-containing protein alpha OS=Rattus norvegicus GN=Sgta PE=1 SV=1 - [SGTA_RAT]	0.92	1.33	0.69	**-0.53**
G3V907	Protein LOC100911356 OS=Rattus norvegicus GN=LOC100360976 PE=4 SV=1 - [G3V907_RAT]	0.30	0.44	0.69	**-0.53**
Q62651	Delta(3,5)-Delta(2,4)-dienoyl-CoA isomerase, mitochondrial OS=Rattus norvegicus GN=Ech1 PE=1 SV=2 - [ECH1_RAT]	0.37	0.55	0.69	**-0.54**
D4A4G0	Uncharacterized protein OS=Rattus norvegicus PE=4 SV=2 - [D4A4G0_RAT]	0.73	1.07	0.68	**-0.55**
F1LRJ9	Selenium-binding protein 1 (Fragment) OS=Rattus norvegicus GN=Selenbp1 PE=2 SV=1 - [F1LRJ9_RAT]	0.72	1.06	0.68	**-0.55**
Q9ER34	Aconitate hydratase, mitochondrial OS=Rattus norvegicus GN=Aco2 PE=1 SV=2 - [ACON_RAT]	0.47	0.70	0.67	**-0.57**
O88989	Malate dehydrogenase, cytoplasmic OS=Rattus norvegicus GN=Mdh1 PE=1 SV=3 - [MDHC_RAT]	1.14	1.71	0.67	**-0.58**
P18163	Long-chain-fatty-acid--CoA ligase 1 OS=Rattus norvegicus GN=Acsl1 PE=1 SV=1 - [ACSL1_RAT]	0.67	1.01	0.67	**-0.59**
Q66HF1	NADH-ubiquinone oxidoreductase 75 kDa subunit, mitochondrial OS=Rattus norvegicus GN=Ndufs1 PE=1 SV=1 - [NDUS1_RAT]	0.75	1.14	0.66	**-0.60**
Q6QI09	ATP synthase gamma chain OS=Rattus norvegicus GN=Atp5c1 PE=2 SV=1 - [Q6QI09_RAT]	0.31	0.47	0.65	**-0.61**
P16409	Myosin light chain 3 OS=Rattus norvegicus GN=Myl3 PE=2 SV=2 - [MYL3_RAT]	2.35	3.69	0.64	**-0.65**
D4A4L5	Protein RGD1563216 OS=Rattus norvegicus GN=RGD1563216 PE=4 SV=1 - [D4A4L5_RAT]	4.18	6.58	0.64	**-0.65**
D4ABR6	Annexin (Fragment) OS=Rattus norvegicus GN=Anxa6 PE=2 SV=2 - [D4ABR6_RAT]	0.21	0.34	0.62	**-0.68**
P53534	Glycogen phosphorylase, brain form (Fragment) OS=Rattus norvegicus GN=Pygb PE=1 SV=3 - [PYGB_RAT]	0.65	1.04	0.62	**-0.69**
G3V6S0	Protein Sptbn1 OS=Rattus norvegicus GN=Sptbn1 PE=4 SV=2 - [G3V6S0_RAT]	0.72	1.16	0.62	**-0.70**
P21913	Succinate dehydrogenase [ubiquinone] iron-sulfur subunit, mitochondrial OS=Rattus norvegicus GN=Sdhb PE=2 SV=2 - [DHSB_RAT]	0.80	1.30	0.62	**-0.70**
P62738	Actin, aortic smooth muscle OS=Rattus norvegicus GN=Acta2 PE=2 SV=1 - [ACTA_RAT]	0.47	0.77	0.62	**-0.70**
P24090	Alpha-2-HS-glycoprotein OS=Rattus norvegicus GN=Ahsg PE=1 SV=2 - [FETUA_RAT]	4.98	8.25	0.60	**-0.73**
Q9WVJ6	Protein Tgm2 OS=Rattus norvegicus GN=Tgm2 PE=2 SV=1 - [Q9WVJ6_RAT]	0.42	0.71	0.60	**-0.74**
P56741	Myosin-binding protein C, cardiac-type OS=Rattus norvegicus GN=Mybpc3 PE=2 SV=2 - [MYPC_RAT]	0.41	0.69	0.59	**-0.76**
P85972	Vinculin OS=Rattus norvegicus GN=Vcl PE=1 SV=1 - [VINC_RAT]	0.57	0.97	0.59	**-0.77**
D3ZWV7	Succinate-semialdehyde dehydrogenase, mitochondrial OS=Rattus norvegicus GN=Aldh5a1 PE=2 SV=1 - [D3ZWV7_RAT]	0.73	1.24	0.59	**-0.77**
D3ZL85	Protein Hccs OS=Rattus norvegicus GN=Hccs PE=4 SV=1 - [D3ZL85_RAT]	0.75	1.29	0.58	**-0.78**
F1LM47	Protein Sucla2 OS=Rattus norvegicus GN=Sucla2 PE=2 SV=1 - [F1LM47_RAT]	0.32	0.54	0.58	**-0.78**
Q5XI77	Annexin OS=Rattus norvegicus GN=Anxa11 PE=2 SV=1 - [Q5XI77_RAT]	1.01	1.75	0.58	**-0.79**
P62909	40S ribosomal protein S3 OS=Rattus norvegicus GN=Rps3 PE=1 SV=1 - [RS3_RAT]	0.64	1.12	0.57	**-0.80**
Q05962	ADP/ATP translocase 1 OS=Rattus norvegicus GN=Slc25a4 PE=1 SV=3 - [ADT1_RAT]	0.42	0.73	0.57	**-0.81**
P08082-2	Isoform Non-brain of Clathrin light chain B OS=Rattus norvegicus GN=Cltb - [CLCB_RAT]	10.98	19.56	0.56	**-0.83**
P11232	Thioredoxin OS=Rattus norvegicus GN=Txn PE=1 SV=2 - [THIO_RAT]	1.89	3.44	0.55	**-0.86**
P97521	Mitochondrial carnitine/acylcarnitine carrier protein OS=Rattus norvegicus GN=Slc25a20 PE=1 SV=1 - [MCAT_RAT]	0.47	0.86	0.55	**-0.87**

Q6P9U0	Protein Serpinb6 OS=Rattus norvegicus GN=Serpinb6 PE=2 SV=1 - [Q6P9U0_RAT]	0.68	1.28	0.53	**-0.92**
B0BN52	Mitochondrial carrier homolog 2 (C. elegans) OS=Rattus norvegicus GN=Mtch2 PE=2 SV=1 - [B0BN52_RAT]	0.55	1.05	0.52	**-0.94**
D4A0T0	Protein Ndufb10 OS=Rattus norvegicus GN=Ndufb10 PE=4 SV=1 - [D4A0T0_RAT]	4.69	8.99	0.52	**-0.94**
P02770	Serum albumin OS=Rattus norvegicus GN=Alb PE=1 SV=2 - [ALBU_RAT]	6.40	12.37	0.52	**-0.95**
P80254	D-dopachrome decarboxylase OS=Rattus norvegicus GN=Ddt PE=1 SV=3 - [DOPD_RAT]	1.06	2.14	0.49	**-1.01**
Q499N5	Acyl-CoA synthetase family member 2, mitochondrial OS=Rattus norvegicus GN=Acsf2 PE=2 SV=1 - [ACSF2_RAT]	0.47	0.96	0.49	**-1.04**
F1LN88	Aldehyde dehydrogenase, mitochondrial OS=Rattus norvegicus GN=Aldh2 PE=2 SV=2 - [F1LN88_RAT]	0.78	1.60	0.48	**-1.05**
G3V7N5	Carnitine O-palmitoyltransferase 2, mitochondrial OS=Rattus norvegicus GN=Cpt2 PE=3 SV=1 - [G3V7N5_RAT]	0.30	0.63	0.48	**-1.06**
D3ZS58	NADH dehydrogenase [ubiquinone] 1 alpha subcomplex subunit 2 OS=Rattus norvegicus GN=Ndufa2 PE=3 SV=1 - [D3ZS58_RAT]	0.68	1.49	0.46	**-1.13**
Q5XI32	F-actin-capping protein subunit beta OS=Rattus norvegicus GN=Capzb PE=1 SV=1 - [CAPZB_RAT]	0.78	1.75	0.44	**-1.17**
P63039	60 kDa heat shock protein, mitochondrial OS=Rattus norvegicus GN=Hspd1 PE=1 SV=1 - [CH60_RAT]	2.13	4.97	0.43	**-1.22**
Q5U302	Catenin (Cadherin associated protein), alpha 1 OS=Rattus norvegicus GN=Ctnna1 PE=2 SV=1 - [Q5U302_RAT]	0.76	1.78	0.42	**-1.23**
G3V7K1	Myomesin 2 OS=Rattus norvegicus GN=Myom2 PE=4 SV=1 - [G3V7K1_RAT]	0.36	0.86	0.41	**-1.28**
Q68FU3	Electron transfer flavoprotein subunit beta OS=Rattus norvegicus GN=Etfb PE=2 SV=3 - [ETFB_RAT]	0.25	0.61	0.40	**-1.31**
P29410-2	Isoform 2 of Adenylate kinase 2, mitochondrial OS=Rattus norvegicus GN=Ak2 - [KAD2_RAT]	1.24	3.24	0.38	**-1.38**
P10111	Peptidyl-prolyl cis-trans isomerase A OS=Rattus norvegicus GN=Ppia PE=1 SV=2 - [PPIA_RAT]	0.87	2.35	0.37	**-1.43**
P17764	Acetyl-CoA acetyltransferase, mitochondrial OS=Rattus norvegicus GN=Acat1 PE=1 SV=1 - [THIL_RAT]	1.21	4.24	0.28	**-1.81**
F1M566	Uncharacterized protein (Fragment) OS=Rattus norvegicus PE=2 SV=2 - [F1M566_RAT]	1.15	4.63	0.25	**-2.01**

* Table includes the redox ratio of individual Cys residues within those proteins. The redox state of selected redox Cys residues labeled with both light ^{12}C-form and heavy ^{13}C-form was calculated using the oxidation ratio (oxidized:reduced).

Table S4. Redox Proteome KEGG pathway enrichment analysis in the *plantaris* muscle of HCR rats.

Term	P-value	Adjusted P-value	log10 (Adj. p value)	
Metabolic pathways_Homo sapiens_hsa01100	8.87159E-29	4.92373E-27	26.31	GPI;ECHS1;NDUFB10;NDUFA10;AKR1B1;PYGM;ENO3;UQCRH;ACAT1;CKMT2;LDHA;ACADL;IMPA1;PGK1;DLAT;HADH;PGM1;IDH3A;NDUFA8;TPI1;PDHA1;MDH1;GOT1;CKM;MDH2;IDH3G;ACSL1;AGL;PGAM2;GOT2;NME2;CMBL;SDHA;PKM;ALDH5A1;IVD;NDUFAB1;UQCRC1;ADSSL1;NDUFS1;SUCLG1;ACO2;ALDOA;DLD;GAPDH
Glycolysis / Gluconeogenesis_Homo sapiens_hsa00010	7.30547E-18	2.70302E-16	15.57	GPI;TPI1;PDHA1;PGAM2;ENO3;LDHA;PKM;PGK1;DLAT;ALDOA;DLD;GAPDH;PGM1
Biosynthesis of amino acids_Homo sapiens_hsa01230	1.43798E-15	3.1923E-14	13.50	TPI1;PKM;GOT1;IDH3G;GOT2;PGAM2;PGK1;ACO2;ALDOA;GAPDH;ENO3;IDH3A
Citrate cycle (TCA cycle) Homo sapiens_hsa00020	1.23975E-16	3.4403E-15	14.46	PDHA1;MDH1;IDH3G;MDH2;SUCLG1;ACO2;DLAT;SDHA;DLD;IDH3A
Pyruvate metabolism_Homo sapiens_hsa00620	2.45337E-13	4.53874E-12	11.34	LDHA;PDHA1;PKM;MDH1;MDH2;GLO1;DLAT;DLD;ACAT1
Oxidative phosphorylation_Homo sapiens_hsa00190	2.72113E-07	2.01808E-06	5.70	NDUFA8;NDUFB10;NDUFAB1;NDUFA10;UQCRC1;NDUFS1;SDHA;UQCRH
Dilated cardiomyopathy_Homo sapiens_hsa05414	2.72713E-07	2.01808E-06	5.70	DES;TPM2;TNNC1;MYL3;ATP2A2;ACTB;MYH7
Non-alcoholic fatty liver disease (NAFLD)_Homo sapiens_hsa04932	7.17938E-07	4.42728E-06	5.35	NDUFA8;NDUFB10;NDUFAB1;NDUFA10;UQCRC1;NDUFS1;SDHA;UQCRH
HIF-1 signaling pathway_Homo sapiens_hsa04066	6.87109E-07	4.42728E-06	5.35	TF;LDHA;PDHA1;PGK1;ALDOA;ENO3;GAPDH
Calcium signaling pathway_Homo sapiens_hsa04020	2.76482E-05	0.000122758	3.91	TNNC1;TNNC2;PPIF;ATP2A2;VDAC1;ATP2A1;SLC25A4
Valine, leucine and isoleucine degradation_Homo sapiens_hsa00280	3.51065E-06	1.77128E-05	4.75	ECHS1;IVD;HADH;DLD;ACAT1
Propanoate metabolism_Homo sapiens_hsa00640	4.3898E-07	3.04543E-06	5.52	LDHA;ECHS1;SUCLG1;DLD;ACAT1
Fatty acid degradation_Homo sapiens_hsa00071	2.26097E-06	1.32088E-05	4.88	ECHS1;ACADL;ACSL1;HADH;ACAT1
Glyoxylate and dicarboxylate metabolism_Homo sapiens_hsa00630	2.17553E-07	1.85757E-06	5.73	MDH1;MDH2;ACO2;DLD;ACAT1
Cysteine and methionine metabolism_Homo sapiens_hsa00270	2.53386E-06	1.40629E-05	4.85	LDHA;GOT1;MDH1;MDH2;GOT2
Fatty acid metabolism_Homo sapiens_hsa01212	3.51065E-06	1.77128E-05	4.75	ECHS1;ACADL;ACSL1;HADH;ACAT1
2-Oxocarboxylic acid metabolism_Homo sapiens_hsa01210	1.42883E-08	1.586E-07	6.80	GOT1;IDH3G;GOT2;ACO2;IDH3A
Tight junction_Homo sapiens_hsa04530	5.94852E-05	0.00024455	3.61	MYLPF;ACTN3;ACTN2;MYH4;ACTB;MYH7
cGMP-PKG signaling pathway_Homo sapiens_hsa04022	0.000163472	0.000585335	3.23	PPIF;ATP2A2;VDAC1;ATP2A1;SLC25A4;MYH7
Glucagon signaling pathway_Homo sapiens_hsa04922	0.000132613	0.000507589	3.29	LDHA;PDHA1;PKM;PGAM2;PYGM
Arrhythmogenic right ventricular cardiomyopathy (ARVC)_Homo sapiens_hsa05412	2.98992E-05	0.000127646	3.89	DES;ACTN3;ACTN2;ATP2A2;ACTB
Adrenergic signaling in cardiomyocytes_Homo sapiens_hsa04261	0.000773694	0.002385555	2.62	TPM2;TNNC1;MYL3;ATP2A2;MYH7
Starch and sucrose metabolism_Homo sapiens_hsa00500	0.000157391	0.000582348	3.23	GPI;AGL;PYGM;PGM1
Central carbon metabolism in cancer_Homo sapiens_hsa05230	0.000315344	0.001060704	2.97	LDHA;PDHA1;PKM;PGAM2
Arginine and proline metabolism_Homo sapiens_hsa00330	0.000100908	0.000400027	3.40	CKMT2;CKM;GOT1;GOT2
Focal adhesion_Homo sapiens_hsa04510	0.003041441	0.008234146	2.08	MYLPF;ACTN3;ACTN2;FLNC;ACTB
Leukocyte transendothelial migration_Homo sapiens_hsa04670	0.002615852	0.007445117	2.13	MYLPF;ACTN3;ACTN2;ACTB
Alanine, aspartate and glutamate metabolism_Homo sapiens_hsa00250	2.42552E-05	0.00011218	3.95	ALDH5A1;GOT1;GOT2;ADSSL1
Purine metabolism_Homo sapiens_hsa00230	0.010649268	0.025697146	1.59	PKM;ADSSL1;NME2;PGM1
Regulation of actin cytoskeleton_Homo sapiens_hsa04810	0.020397858	0.046207392	1.34	MYLPF;ACTN3;ACTN2;ACTB

Adherens junction_Homo sapiens_hsa04520	0.005589132	0.014427759	1.84	ACTN3;ACTN2;ACTB
Lysine degradation_Homo sapiens_hsa00310	0.002057487	0.006010029	2.22	ECHS1;HADH;ACAT1
Butanoate metabolism_Homo sapiens_hsa00650	9.73478E-06	4.69809E-05	4.33	ALDH5A1;ECHS1;HADH;ACAT1
Phagosome_Homo sapiens_hsa04145	0.039052418	0.080274415	1.10	CTSL;TUBB4A;ACTB
Proteoglycans in cancer_Homo sapiens_hsa05205	0.076090018	0.136225677	0.87	CTSL;FLNC;ACTB
Viral carcinogenesis_Homo sapiens_hsa05203	0.077845166	0.137155768	0.86	PKM;ACTN3;ACTN2
Apoptosis_Homo sapiens_hsa04210	0.147871812	0.231179875	0.64	CTSL;ACTB
Amoebiasis_Homo sapiens_hsa05146	0.084924115	0.145024258	0.84	ACTN3;ACTN2
Thyroid hormone signaling pathway_Homo sapiens_hsa04919	0.112114519	0.183010465	0.74	ATP2A2;ACTB
RNA degradation_Homo sapiens_hsa03018	0.053872433	0.10310069	0.99	HSPA9;ENO3
Inositol phosphate metabolism_Homo sapiens_hsa00562	0.046608457	0.09238462	1.03	TPI1;IMPA1
Systemic lupus erythematosus_Homo sapiens_hsa05322	0.139545207	0.222008618	0.65	ACTN3;ACTN2
Influenza A_Homo sapiens_hsa05164	0.208440812	0.304433292	0.52	VDAC1;ACTB
Pancreatic secretion_Homo sapiens_hsa04972	0.079196081	0.137355702	0.86	ATP2A2;ATP2A1
Salmonella infection_Homo sapiens_hsa05132	0.065455509	0.119107566	0.92	FLNC;ACTB
Peroxisome_Homo sapiens_hsa04146	0.061507369	0.113788633	0.94	ACSL1;SOD2
MAPK signaling pathway_Homo sapiens_hsa04010	0.712840724	0.712840724	0.15	FLNC
Rap1 signaling pathway_Homo sapiens_hsa04015	0.643448548	0.649298081	0.19	ACTB
PPAR signaling pathway_Homo sapiens_hsa03320	0.044274388	0.089353766	1.05	ACADL;ACSL1
cAMP signaling pathway_Homo sapiens_hsa04024	0.621799554	0.63320872	0.20	ATP2A2
HTLV-I infection_Homo sapiens_hsa05166	0.356620209	0.41668256	0.38	VDAC1;SLC25A4
Tuberculosis_Homo sapiens_hsa05152	0.580739124	0.596870766	0.22	HSPA9
Jak-STAT signaling pathway_Homo sapiens_hsa04630	0.53754346	0.557638543	0.25	FHL1
Hippo signaling pathway_Homo sapiens_hsa04390	0.526073658	0.55613501	0.25	ACTB
Oxytocin signaling pathway_Homo sapiens_hsa04921	0.53754346	0.557638543	0.25	ACTB
Insulin signaling pathway_Homo sapiens_hsa04910	0.492438881	0.525583806	0.28	PYGM
Tryptophan metabolism_Homo sapiens_hsa00380	0.000959291	0.002877873	2.54	ECHS1;HADH;ACAT1
FoxO signaling pathway_Homo sapiens_hsa04068	0.477310604	0.514383272	0.29	SOD2
Platelet activation_Homo sapiens_hsa04611	0.448407362	0.490901859	0.31	ACTB
Lysosome_Homo sapiens_hsa04142	0.451099006	0.490901859	0.31	CTSL
Insulin resistance_Homo sapiens_hsa04931	0.412204199	0.462168345	0.34	PYGM
Toxoplasmosis_Homo sapiens_hsa05145	0.437509538	0.485635587	0.31	PPIF
Viral myocarditis_Homo sapiens_hsa05416	0.033307124	0.069756429	1.16	ACTB;MYH7
Pyrimidine metabolism_Homo sapiens_hsa00240	0.40059879	0.453739446	0.34	NME2
Longevity regulating pathway - mammal_Homo sapiens_hsa04211	0.367501619	0.424923747	0.37	SOD2
Phosphatidylinositol signaling system_Homo sapiens_hsa04070	0.379741126	0.434549124	0.36	IMPA1
Rheumatoid arthritis_Homo sapiens_hsa05323	0.355023129	0.41668256	0.38	CTSL
Gap junction_Homo sapiens_hsa04540	0.348692826	0.416181761	0.38	TUBB4A
Pathogenic Escherichia coli infection_Homo sapiens_hsa05130	0.029269358	0.062478821	1.20	TUBB4A;ACTB
Chemical carcinogenesis_Homo sapiens_hsa05204	0.329330647	0.397344584	0.40	GSTM3

Bacterial invasion of epithelial cells_Homo sapiens_hsa05100	0.316107154	0.385581254	0.41	ACTB
Gastric acid secretion_Homo sapiens_hsa04971	0.302625676	0.377432023	0.42	ACTB
Adipocytokine signaling pathway_Homo sapiens_hsa04920	0.288881233	0.372858336	0.43	ACSL1
Antigen processing and presentation_Homo sapiens_hsa04612	0.312761164	0.385581254	0.41	CTSL
Metabolism of xenobiotics by cytochrome P450_Homo sapiens_hsa00980	0.299214415	0.377418183	0.42	GSTM3
Thyroid hormone synthesis_Homo sapiens_hsa04918	0.292342273	0.372988417	0.43	GPX1
Drug metabolism - cytochrome P450_Homo sapiens_hsa00982	0.285403441	0.372703318	0.43	GSTM3
Longevity regulating pathway - multiple species_Homo sapiens_hsa04213	0.267760381	0.358089184	0.45	SOD2
Glutathione metabolism_Homo sapiens_hsa00480	0.026380646	0.0574167	1.24	GSTM3;GPX1
Shigellosis_Homo sapiens_hsa05131	0.271323144	0.358534154	0.45	ACTB
Amino sugar and nucleotide sugar metabolism_Homo sapiens_hsa00520	0.022722972	0.050444997	1.30	GPI;PGM1
Glycerolipid metabolism_Homo sapiens_hsa00561	0.249686322	0.342162737	0.47	AKR1B1
Arachidonic acid metabolism_Homo sapiens_hsa00590	0.260583055	0.352740477	0.45	GPX1
Amyotrophic lateral sclerosis (ALS)_Homo sapiens_hsa05014	0.219845257	0.305035293	0.52	GPX1
Vibrio cholerae infection_Homo sapiens_hsa05110	0.219845257	0.305035293	0.52	ACTB
Malaria_Homo sapiens_hsa05144	0.212203291	0.305035293	0.52	HBB
Type II diabetes mellitus_Homo sapiens_hsa04930	0.208354574	0.304433292	0.52	PKM
Mineral absorption_Homo sapiens_hsa04978	0.219845257	0.305035293	0.52	TF
Pentose phosphate pathway_Homo sapiens_hsa00030	0.000368748	0.001203854	2.92	GPI;ALDOA;PGM1
Glycine, serine and threonine metabolism_Homo sapiens_hsa00260	0.016110299	0.037255067	1.43	PGAM2;DLD
Fat digestion and absorption_Homo sapiens_hsa04975	0.18088752	0.27133128	0.57	GOT2
Fructose and mannose metabolism_Homo sapiens_hsa00051	0.000495297	0.0015708	2.80	TPI1;AKR1B1;ALDOA
African trypanosomiasis_Homo sapiens_hsa05143	0.156595108	0.241417459	0.62	HBB
Galactose metabolism_Homo sapiens_hsa00052	0.009270658	0.022867624	1.64	AKR1B1;PGM1
Tyrosine metabolism_Homo sapiens_hsa00350	0.012483195	0.029481588	1.53	GOT1;GOT2
Pentose and glucuronate interconversions_Homo sapiens_hsa00040	0.160692793	0.244341096	0.61	AKR1B1
beta-Alanine metabolism_Homo sapiens_hsa00410	0.140005435	0.222008618	0.65	ECHS1
Fatty acid elongation_Homo sapiens_hsa00062	0.00649483	0.016384685	1.79	ECHS1;HADH
Proximal tubule bicarbonate reclamation_Homo sapiens_hsa04964	0.105851209	0.175365435	0.76	MDH1
Terpenoid backbone biosynthesis_Homo sapiens_hsa00900	0.10148863	0.170685423	0.77	ACAT1
Arginine biosynthesis_Homo sapiens_hsa00220	0.00417867	0.011043627	1.96	GOT1;GOT2
Phenylalanine metabolism_Homo sapiens_hsa00360	0.00301948	0.008234146	2.08	GOT1;GOT2
Fatty acid biosynthesis_Homo sapiens_hsa00061	0.061265331	0.113788633	0.94	ACSL1
Synthesis and degradation of ketone bodies_Homo sapiens_hsa00072	0.047465511	0.092432837	1.03	ACAT1

Phenylalanine, tyrosine and tryptophan biosynthesis_Homo sapiens_hsa00400	0.000230607	0.00079992	3.10	GOT1;GOT2

Table S5. Redox Proteome KEGG pathway enrichment analysis in the heart of HCR rats.

Term	P-value	Adjusted P-value	log10 (Adj. p value)	Genes
Huntington's disease_Homo sapiens_hsa05016	3.83939E-09	2.45721E-07	6.61	NDUFB10;NDUFA2;CLTB;ATP5C1;NDUFS1;SDHB;SLC25A4;TGM2
Metabolic pathways_Homo sapiens_hsa01100	4.63571E-07	9.88952E-06	5.00	PYGB;MDH1;NDUFB10;ACSL1;NDUFA2;AK2;ATP5C1;SDHB;ACAT1;SUCLA2;ALDH5A1;ALDH2;NDUFS1
Parkinson's disease_Homo sapiens_hsa05012	3.62506E-07	9.88952E-06	5.00	NDUFB10;NDUFA2;ATP5C1;NDUFS1;SDHB;SLC25A4
Fatty acid degradation_Homo sapiens_hsa00071	1.75725E-06	2.8116E-05	4.55	ALDH2;CPT2;ACSL1;ACAT1
Oxidative phosphorylation_Homo sapiens_hsa00190	6.58232E-06	8.42536E-05	4.07	NDUFB10;NDUFA2;ATP5C1;NDUFS1;SDHB
Alzheimer's disease_Homo sapiens_hsa05010	2.04372E-05	0.000217997	3.66	NDUFB10;NDUFA2;ATP5C1;NDUFS1;SDHB
Pyruvate metabolism_Homo sapiens_hsa00620	6.9558E-05	0.000536662	3.27	ALDH2;MDH1;ACAT1
Carbon metabolism_Homo sapiens_hsa01200	7.54681E-05	0.000536662	3.27	SUCLA2;MDH1;SDHB;ACAT1
Non-alcoholic fatty liver disease (NAFLD)_Homo sapiens_hsa04932	0.000230963	0.001343784	2.87	NDUFB10;NDUFA2;NDUFS1;SDHB
Citrate cycle (TCA cycle)_Homo sapiens_hsa00020	2.89826E-05	0.000264984	3.58	SUCLA2;MDH1;SDHB
Fatty acid metabolism_Homo sapiens_hsa01212	0.000120426	0.000770729	3.11	CPT2;ACSL1;ACAT1
Bacterial invasion of epithelial cells_Homo sapiens_hsa05100	0.000508147	0.002710115	2.57	CLTB;CTNNA1;VCL
Valine, leucine and isoleucine degradation_Homo sapiens_hsa00280	0.004150893	0.015626891	1.81	ALDH2;ACAT1
Tryptophan metabolism_Homo sapiens_hsa00380	0.002899404	0.011597616	1.94	ALDH2;ACAT1
Lysine degradation_Homo sapiens_hsa00310	0.004854972	0.017262123	1.76	ALDH2;ACAT1
Propanoate metabolism_Homo sapiens_hsa00640	0.001862438	0.007946403	2.10	SUCLA2;ACAT1
Adherens junction_Homo sapiens_hsa04520	0.009619374	0.030781996	1.51	CTNNA1;VCL
Butanoate metabolism_Homo sapiens_hsa00650	0.001426549	0.006521366	2.19	ALDH5A1;ACAT1
Dilated cardiomyopathy_Homo sapiens_hsa05414	0.013979873	0.04066872	1.39	MYBPC3;MYL3
PPAR signaling pathway_Homo sapiens_hsa03320	0.008407741	0.028320813	1.55	CPT2;ACSL1
Hypertrophic cardiomyopathy (HCM)_Homo sapiens_hsa05410	0.011983291	0.036520507	1.44	MYBPC3;MYL3
Amoebiasis_Homo sapiens_hsa05146	0.017063339	0.047480594	1.32	VCL;SERPINB6
Glyoxylate and dicarboxylate metabolism_Homo sapiens_hsa00630	0.001426549	0.006521366	2.19	MDH1;ACAT1
Leukocyte transendothelial migration_Homo sapiens_hsa04670	0.023265854	0.059560587	1.23	CTNNA1;VCL
Endocytosis_Homo sapiens_hsa04144	0.094577763	0.160613747	0.79	CAPZB;CLTB
Glucagon signaling pathway_Homo sapiens_hsa04922	0.183484991	0.239653866	0.62	PYGB
Insulin resistance_Homo sapiens_hsa04931	0.196525809	0.251553035	0.60	PYGB
Endometrial cancer_Homo sapiens_hsa05213	0.098988478	0.16244263	0.79	CTNNA1
Vascular smooth muscle contraction_Homo sapiens_hsa04270	0.214125852	0.268706952	0.57	ACTA2
Lysosome_Homo sapiens_hsa04142	0.218860259	0.269366472	0.57	CLTB

Glycolysis / Gluconeogenesis_Homo sapiens_hsa00010	0.125721422	0.182867523	0.74	ALDH2
Starch and sucrose metabolism_Homo sapiens_hsa00500	0.106194192	0.169910707	0.77	PYGB
Endocrine and other factor-regulated calcium reabsorption_Homo sapiens_hsa04961	0.089901652	0.159825159	0.80	CLTB
Adipocytokine signaling pathway_Homo sapiens_hsa04920	0.130974433	0.18627475	0.73	ACSL1
Ribosome_Homo sapiens_hsa03010	0.24058897	0.283513673	0.55	RPS3
Glycerolipid metabolism_Homo sapiens_hsa00561	0.111561575	0.174144897	0.76	ALDH2
Adrenergic signaling in cardiomyocytes_Homo sapiens_hsa04261	0.257247033	0.293996609	0.53	MYL3
Insulin signaling pathway_Homo sapiens_hsa04910	0.243644562	0.283513673	0.55	PYGB
Tight junction_Homo sapiens_hsa04530	0.243644562	0.283513673	0.55	CTNNA1
Cardiac muscle contraction_Homo sapiens_hsa04260	0.144832472	0.197218686	0.71	MYL3
cGMP-PKG signaling pathway_Homo sapiens_hsa04022	0.285184698	0.314686563	0.50	SLC25A4
Calcium signaling pathway_Homo sapiens_hsa04020	0.3037068	0.32395392	0.49	SLC25A4
Peroxisome_Homo sapiens_hsa04146	0.153384079	0.204512105	0.69	ACSL1
Arrhythmogenic right ventricular cardiomyopathy (ARVC)_Homo sapiens_hsa05412	0.137930603	0.191903448	0.72	CTNNA1
Hippo signaling pathway_Homo sapiens_hsa04390	0.26470059	0.29720768	0.53	CTNNA1
Purine metabolism_Homo sapiens_hsa00230	0.298058107	0.323317269	0.49	AK2
Shigellosis_Homo sapiens_hsa05131	0.122202228	0.181882386	0.74	VCL
Arginine and proline metabolism_Homo sapiens_hsa00330	0.095364413	0.160613747	0.79	ALDH2
Focal adhesion_Homo sapiens_hsa04510	0.333991262	0.350417062	0.46	VCL
Regulation of actin cytoskeleton_Homo sapiens_hsa04810	0.349964719	0.361253903	0.44	VCL
HTLV-I infection_Homo sapiens_hsa05166	0.405400645	0.411835576	0.39	SLC25A4
Synaptic vesicle cycle_Homo sapiens_hsa04721	0.118669224	0.180829294	0.74	CLTB
Cysteine and methionine metabolism_Homo sapiens_hsa00270	0.086241957	0.157699579	0.80	MDH1
Pathways in cancer_Homo sapiens_hsa05200	0.551917178	0.551917178	0.26	CTNNA1
Porphyrin and chlorophyll metabolism_Homo sapiens_hsa00860	0.080725498	0.151953878	0.82	HCCS
Alanine, aspartate and glutamate metabolism_Homo sapiens_hsa00250	0.06772718	0.13497211	0.87	ALDH5A1
Pentose and glucuronate interconversions_Homo sapiens_hsa00040	0.069594994	0.13497211	0.87	ALDH2
beta-Alanine metabolism_Homo sapiens_hsa00410	0.060219295	0.124323706	0.91	ALDH2
Proximal tubule bicarbonate reclamation_Homo sapiens_hsa04964	0.045026255	0.102917155	0.99	MDH1
Ascorbate and aldarate metabolism_Homo sapiens_hsa00053	0.05265247	0.11232527	0.95	ALDH2
Histidine metabolism_Homo sapiens_hsa00340	0.046938402	0.103588197	0.98	ALDH2
Terpenoid backbone biosynthesis_Homo sapiens_hsa00900	0.043110368	0.10218754	0.99	ACAT1
Fatty acid biosynthesis_Homo sapiens_hsa00061	0.025697895	0.063256356	1.20	ACSL1
Synthesis and degradation of ketone bodies_Homo sapiens_hsa00072	0.019825378	0.052867674	1.28	ACAT1
